# Immune-mediated changes in actinic keratosis following topical treatment with imiquimod 5% cream

**DOI:** 10.1186/1479-5876-5-7

**Published:** 2007-01-26

**Authors:** Abel Torres, Leslie Storey, Makala Anders, Richard L Miller, Barbara J Bulbulian, Jizhong Jin, Shalini Raghavan, James Lee, Herbert B Slade, Woubalem Birmachu

**Affiliations:** 1 Dermatology Office, Loma Linda University Medical Center, Loma Linda, California, USA; 2 Pharmacology, 3M Pharmaceuticals, St Paul, Minnesota, USA; 3 Medical & Scientific Affairs, 3M Pharmaceuticals, St Paul, Minnesota, USA

## Abstract

**Background:**

The objective of this study was to identify the molecular processes responsible for the anti-lesional activity of imiquimod in subjects with actinic keratosis using global gene expression profiling.

**Methods:**

A double-blind, placebo-controlled, randomized study was conducted to evaluate gene expression changes in actinic keratosis treated with imiquimod 5% cream. Male subjects (N = 17) with ≥ 5 actinic keratosis on the scalp applied placebo cream or imiquimod 3 times a week on nonconsecutive days for 4 weeks. To elucidate the molecular processes involved in actinic keratosis lesion regression by imiquimod, gene expression analysis using oligonucleotide arrays and real time reverse transcriptase polymerase chain reaction were performed on shave biopsies of lesions taken before and after treatment.

**Results:**

Imiquimod modulated the expression of a large number of genes important in both the innate and adaptive immune response, including increased expression of interferon-inducible genes with known antiviral, anti-proliferative and immune modulatory activity, as well as various Toll-like receptors. In addition, imiquimod increased the expression of genes associated with activation of macrophages, dendritic cells, cytotoxic T cells, and natural killer cells, as well as activation of apoptotic pathways.

**Conclusion:**

Data suggest that topical application of imiquimod stimulates cells in the skin to secrete cytokines and chemokines that lead to inflammatory cell influx into the lesions and subsequent apoptotic and immune cell-mediated destruction of lesions.

## Background

Actinic keratosis (AK) are common, cutaneous, precancerous neoplasms appearing as rough, dry, scaly lesions that occur primarily on the sun-exposed skin of middle-aged and elderly people [1-3]. Although the exact mechanism of pathogenesis of AK development is unknown, part of the pathogenesis may involve suppression of the immune response against dysplastic cells [4]. It is believed that prolonged ultraviolet exposure changes the immune surveillance mechanism of the skin, contributing to the tolerance of tumor cells [5]. If left untreated, AK can progress to squamous cell carcinoma, a locally aggressive and occasionally metastatic tumor type [6]. Standard treatment of AK includes various types of surgical and chemical treatments [7,8], which are often associated with scarring and infection, and may not address sub clinical lesions [8].

Toll-like receptors (TLR) are pattern recognition receptors that detect pathogen-associated molecular patterns (PAMPs) and play key roles in the activation of innate and adaptive immune responses [9,10]. Currently, 10 human TLRs have been identified. The natural ligands for all but TLR10 have also been identified [9]. Toll-like receptors are primarily expressed on immune cells such as monocytes, dendritic cells (DCs), and lymphocytes [11], but some TLRs are also expressed on nonimmune cells, including endothelial cells, epithelial cells, and keratinocytes [12].

The role of TLRs in the pathogenesis and treatment of dermatological diseases is increasingly recognized [13]. Imiquimod, a member of a class of drugs termed immune response modifiers has been shown to be a selective TLR7 agonist [[14,15], and unpublished internal data]. Imiquimod is the first TLR-agonist pharmaceutical product approved for human use, and is indicated for the topical treatment of external genital and perianal warts caused by human papilloma virus [16]. Recently, the approved indications have been expanded to include treatment of AK [17] and superficial basal cell carcinoma [18-20].

The antiviral and anti-tumor activity of imiquimod is believed to be due to the activation of the innate immune response, specifically activation of antigen-presenting cells such as monocytes, macrophages and plasmacytoid and myeloid DCs to induce interferon alpha (IFNα) and other cytokines and chemokines [21,15]. Imiquimod also enhances co stimulatory molecule expression important for triggering an adaptive immune response [15]. Topical application of the drug has been shown to induce IFNα and interleukin 6 (IL6) in AK lesions and external genital warts [22,23]. Imiquimod and the chemically related immune response modifier resiquimod have also shown potent vaccine adjuvant effects in mice and man [23-27]. Even though the immune-modulatory activity of imiquimod is well established, the precise molecular changes responsible for the antilesional activity of topically applied imiquimod in AK is not fully understood.

The objective of this study was to explore the molecular processes responsible for the antilesional activity of imiquimod in subjects with actinic keratosis using global gene expression profiling.

## Methods and Materials

### Institutional review board/informed consent

This study was conducted at Loma Linda University School of Medicine/Medical Center, Department of Internal Medicine, Division of Dermatology, Loma Linda, California. The study protocol, subject informed consent documents, and subject information documents were submitted to and received approval from the study center's Institution Review Board. This study was conducted according to the Code of Federal Regulations of the United States Food and Drug Administration (21 CFR Part 56, Institutional Review Boards, and Part 50, Protection of Human Subjects) and the International Conference on Harmonization Edition 6, Guideline for Good Clinical Practice.

### Study Conduct

This was a phase II, double-blind, placebo-controlled, randomized parallel group study. Randomized subjects had at least 5 clinically visible AK lesions within a 25-cm^2 ^area on the balding scalp. Subjects were randomized to imiquimod or placebo cream in a 3:1 ratio and applied study cream to the treatment area 3 times per week for 4 weeks. Study cream was applied prior to normal sleeping hours and remained on the skin for approximately 8 hours before it was removed. Safety evaluations were made at all treatment and post treatment visits, and included monitoring of adverse events and local skin reactions, as well as photographing the treatment area and reviewing any concomitant medications.

At the screening visit, AK lesions were assessed clinically and by confocal microscopy and a representative lesion confirmed by histology. Because AK lesions are in general small in size, histology and gene expression analysis could not be performed on the same biopsy. Therefore, confocal microscopy was performed to establish a correlation of the confocal images and their respective non-sun exposed non-lesional skin, sun exposed non-lesional skin, actinic keratoses lesions, and squamous cell carcinoma. All subjects with lesions histologically identified as having a degree of dysplesia suggestive of squamous cell carcinoma were disqualified from the study. All sites identified as AK lesions were marked and a plastic template of their locations made for exact identification at a later time. Thereafter, changes in AK lesions due to treatment with imiquimod were assessed clinically and by confocal microscopy. Lesions were scored as cleared if the skin exhibited normal epidermis as assessed clinically and by confocal microscopy. Assessment of lesion regression and the results of the confocal microscopy as they relate to aberrant gene expression in AK are discussed in a manuscript submitted for publication (Torres et al, 2006. Micro Array Analysis of Aberrant Gene Expression in Actinic Keratosis: Effect of Imiquimod 5% Cream).

At the treatment initiation visit, a shave biopsy was taken for gene expression analysis from an untreated AK lesion, from a sun-exposed non-lesional site on the head and from a non-lesional sun-unexposed site from under the arm area. An additional biopsy was taken at each subsequent study visit, (treatment period weeks 1, 2, and 4) and at 4 weeks post treatment of either a remaining AK, or if no AKs were present, of nonlesional skin at a previously identified lesional site. Thus each biopsy was of a different AK lesion. In an effort to standardize the amount of tissue that was removed at each biopsy, the same size punch was used to score the skin surrounding all the lesions to be biopsied for gene expression studies with an attempt to shave-biopsy the lesion at the papillary dermis level. Biopsies were taken 8 hr to 16 hr after the third treatment of the treatment period. The nonlesional, sun-unexposed site biopsy was used to establish a baseline for comparison of gene expression changes in AK before and after treatment with imiquimod. Shave biopsies were immediately immersed in RNALater (Ambion, Austin, Texas), equilibrated at room temperature for 1 hour, kept at 4°C for 24 hours, and then stored at -20°C prior to RNA extraction.

### RNA extraction and purification

Total RNA from the biopsy samples was extracted and purified using Qiagen RNeasy Mini Kit Protocol for the Isolation of Total RNA from Heart, Muscle and Skin Tissue (Qiagen, Valencia, California) according to manufacturer's instructions. RNA yield varied from 1.5 to 14 υg. The purity of the RNA was determined by the 260 nm/280 nm absorbance ratio. The median 260 nm/280 nm ratio value for the 119 samples was 2.0 (range 1.9 to 2.2). RNA integrity was determined using the Agilent 2100 Bioanalyzer and the RNA 6000 Nano Assay (Agilant Technologies, Palo Alto, CA). All samples gave 28S/18S ratio between 2 and 3 indicating good quality RNA.

### Micro array analysis

Samples for micro array analysis were prepared by 2 rounds of linear target amplification according to the Affymetrix instructions for eukaryotic small sample preparation [28,29]. Briefly, double-stranded cDNA was synthesized from 100 ng of total RNA with oligo(dT)24T7 primer (Affymetrix, Santa Clara, California), followed by 2 cycles of *in vitro *transcription of cRNA. The first cycle of *invitro *transcription was performed using a T7 polymerase (MEGAscript T7 Kit, Ambion, Austin, Texas) and the second cycle using Enzo BioArray High Yield RNA Transcript Labeling Kit (Affymetrix). The median value for the biotinylated cRNA yield over the 119 samples was 90 (range 42 to 121). The biotinylated cRNA was hybridized to Affymetrix U133A and U133B GeneChip arrays containing 22,253 probes sets each. Each array was hybridized for 16 hours, washed, then stained with streptavidin-phycoerythrin conjugate and scanned according to manufacturer's instructions. Images were analyzed using Micro Array Suite Version 5 (MAS5). Chips were normalized to a global average intensity of 150 to allow chip-to-chip comparison. The quality of the images was ascertained by monitoring the noise, background, percent transcript present, and the 3'/5' ratio for the housekeeping gene glyceraldehyde-3-phosphate dehydrogenase (GAPDH) which ranged from 1.5 to 5 and beta actin which ranged from 7 to 24. These values are similar to those reported for double amplification protocols [29,30].

### TaqMan™ real time reverse transcriptase polymerase chain reaction

TaqMan real time RT-PCR was performed for a number of the genes to confirm the micro array results. cDNA was reverse transcribed from total RNA using Invitrogen Superscript First-Strand Synthesis System for RT-PCR (Invitrogen, Carlsbad, California). Real time RT-PCR was performed using the Applied Biosystems 7900HT™ sequence detection instrument (Applied Biosystems, Foster City, California), and TaqMan low density custom array micro fluidic cards (Applied Biosystems, Foster City, California) as described previously[31]. The micro fluidic cards consisted of 8 ports with 24 different TaqMan primer pair/probe sets arrayed in duplicate in a 384-well micro plate footprint. Each well contained a gene-specific forward and reverse primer, as well as a gene-specific probe, which is labeled at the 5' position with 6FAM (areporter dye) and at the 3' position with minor groove binder/non-fluorescent quencher. Samples were mixed with TaqMan Universal PCR Master Mix (Applied Biosystems, Foster City, California), applied to each port of the card, and analyzed by PCR on the 7900HT instrument using Applied Biosystems Sequence Detection System 2.0 software according to the manufacturer's instructions. A total of 46 selected genes associated with the Toll-like receptor pathway, apoptosis, cell cycle, and immune cell infiltration were analyzed using the TaqMan arrays. The housekeeping gene glyceraldehyde-3-phosphate dehydrogenase (GAPDH) was used to normalize each sample. The TLR copy number was calculated for 2 ng of RNA.

### Analysis of affymetrix gene chip data

Signals from the gene chip images of biopsy samples for untreated normal skin obtained from sun-unexposed sites were used as a control for the calculation of changes in expression in samples of pretreatment AK lesions and AK lesions during and after treatment (treatment weeks 1, 2, and 4 and 4 weeks post treatment). The statistical algorithm in MAS5 evaluates the image for the expression signal, the absent/present call, and the p-value associated with the signal. It also evaluates the fold change of the sample relative to the designated control sample expressed as the signal log2 ratio, the p-value associated with the fold change, and the direction of change (increased, I; decreased, D; or no call, NC). Using the Affymetrix data mining tool software, the expression data from MAS5 were filtered on the basis of specific criteria to identify differentially expressed genes. A given gene was retained if 1sample from the series passed the following criteria: signal detection p-value ≤ 0.01, signal log2 ratio ≤ -2 or ≥ 2, and a change call designation of 'increased' (I) or 'decreased' (D). A total of 1682 genes passed these criteria.

**Figure 1:**
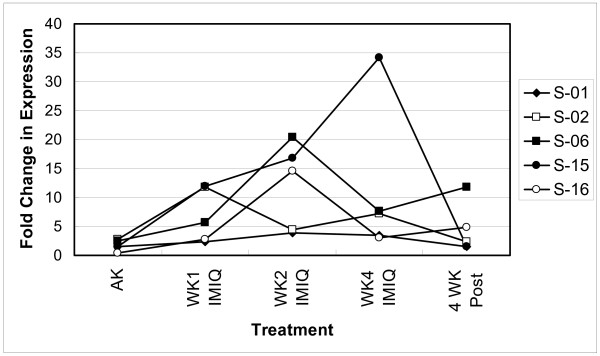
Variation in the temporal expression of IRF7 during treatment with imiquimod as determined by real time RT-PCR. 'AK,' designates pretreatment AK lesions. 'WK1 IMIQ', 'WK2 IMIQ' and 'WK4 IMIQ' designate treatment times week 1, 2 and 4. '4 WK Post designates' 4 weeks post end of treatment (WK4 treatment). Fold change was calculated with respect to sun-unexposed, non-lesional skin. S-01, S-02, S-06, S-15 and S-16 are samples from subjects 1, 2, 6, 15 and 16.

There was a large subject-to-subject variation in the magnitude of changes in expression, as well as the temporal pattern of expression (subject-specific variation in magnitude of response with treatment time) of the 1682 genes. This variation is exemplified in Figure 1, which shows the variation in the temporal expression of IRF7 for selected subjects. Peak expression varied from week 1-treatment to week 4-treatment depending on the subject. This variation may be due to the inherent differences in the responses of subjects and (or) due to variation in the time from treatment to biopsy (range 8 to 16 hr). In order to identify differentially expressed genes in the treatment group without regard to variation in temporal response, the data were reduced in the following manner. The fold change values for samples in the week 1, week 2 and week 4 treatment groups for the 1682 genes for each subject were compared to identify the maximum change in expression (increased or decreased) due to imiquimod or placebo treatment. This value was designated as the treatment-response fold change for imiquimod or placebo and used in the ANOVA described in the Statistical Analysis section.

Cluster analysis was performed with Spotfire DecisionSite-8.1 for Functional Genomics (Spotfire Inc, Somerville, Massachusetts), using the Unweighted Pair-Group Method with Arithmetic mean (UPGMA) and the Euclidean similarity measure. Functional categorization of genes was based on gene ontology analysis using the Ontology Browser in Spotfire and gene descriptions at the National Center for Biotechnology Information (NCBI) website [32]. The ontology browser calculates a Fisher's Exact Test p-value, which reflects the chance that the gene ontology category is represented by random chance [33]. P-value < 0.05 is considered significant.

### Statistical analysis

The natural log of the fold change (with respect to sun-unexposed normal skin) from the Affymetrix gene expression and the real time RT-PCR experiments were used in an ANOVA to determine statistically significant changes in expression between sample groups. Due to the small sample size of the vehicle group, 4 subjects, compared to 13 subjects in the imiquimod-treated group, a one way ANOVA comparing vehicle-treated subjects to imiquimod-treated subjects is not expected to yield a reliable determination of imiquimod-response genes. Therefore, a 2-way ANOVA using a blocking factor to account for repeat observations on the same subject was used to compare: (1) the fold change for pretreatment AK (n = 13) and the fold change during treatment with imiquimod (n = 13), and (2) the fold change for pretreatment AK (n = 13) and the fold change for samples taken 4 weeks after the last imiquimod treatment (n = 13). The 'during treatment' fold change is the maximum response fold change value (decreased or increased) selected from week 1, week 2 and week 4 treatment fold changes. Differences between sample groups were considered significant if the p-value for the ANOVA was < 0.05.

## Results and Discussion

### Demographics and response to treatment

Seventeen white males were randomized to receive treatment (13 to imiquimod and 4 to placebo). The mean age was 75 years (range, 62 to 89 years). All 17 subjects in the study completed the treatment and post treatment portions of the study. The median number of AK lesions at baseline was 10 per subject (range, 6 to 13 lesions). Because of the short duration of the follow-up period (4 weeks for the post treatment period), efficacy was not measured in this study. However, clinical clearance was observed in 25% of the imiquimod-treated subjects 4 weeks after the end of treatment. Imiquimod-treated subjects 1, 2, 4, and 6 and placebo-treated subject 7 were assessed as having clinical clearance of lesions 4 weeks post treatment as determined by return of the lesional site to normal skin. The complete clearance rate in a study where AK subjects were treated for 16 weeks, with a post treatment period of 8 weeks, was 57% [17].

### Analysis of global gene expression using Affymetix GeneChips: Gene Ontology classification

A 2-way analysis of variance (ANOVA) was performed comparing the treatment response gene expression fold change (the maximum response value from week 1, week 2 and week 4 treatments) of samples from the imiquimod-treated subjects to the gene expression fold change values of their respective pretreatment AK samples. This comparison resulted in 530 unique genes that had p-values < 0.05 and a median fold change from pretreatment AK <-2 for suppressed genes and >2 for induced genes. Data for the 530 genes is documented in [Additional file 1]. Two-way ANOVA comparing pretreatment AK samples and samples taken 4 weeks after the last imiquimod treatment resulted in 111 unique genes that differentiated the 2 groups with a p-value < 0.05 [see Additional file 1]. The expression of the rest of the genes returned to basal levels 4 weeks post treatment. Of the total number of differentially regulated genes during imiquimod treatment or 4 weeks post treatment, 87% were up-regulated and 13% were down-regulated.

Table 1 summarizes the gene ontology classification of imiquimod-modulated genes from the Affymetrix analysis. P-values for representation of ontology categories are given for categories with p-values < 0.05. Of the 530 unique genes whose expression was modulated by treatment, 436 genes had some annotation in the ontology database. Of these, 106 were annotated to the immune response category, 123 to signal transduction, 72 to receptor activity, and 25 to inflammatory response. Specific genes in several molecular function categories are also listed. Other ontology categories that were represented in the data but had p-values >0.05 for representation in the gene ontology category included metabolism (148 genes), development (37 genes) and regulation of transcription (24 genes). The ontology analysis shows that imiquimod treatment of AK results in global gene expression changes impacting various cellular processes, with immune response and signal transduction being the 2 major processes represented.

**Table 1:** Gene Ontology Classification of Imiquimod-Induced Genes in AK Lesions

**Ontology**	**Genes in the Data**^1^	**Annotated Genes**^2^	**P-value**^3^	**Increased Expression**	**Decreased Expression**	**Description of Processes and Specific Genes**
Total	436	17479		369	67	
Response to stimuli	137	2475	2.13E-19	125	12	defense response, response to external biotic stimuli including, pest and pathogens, antimicrobial, anti-fungal, anti-viral response
Defense response	105	1174	1.54E-40	102	3	immune response, inflammatory response
Immune response	106	1020	3.05E-40	104	2	cellular defense response, humoral defense response, antigen binding, pattern binding, cytokine synthesis, chemokine synthesis, antigen presentation processing, inflammatory response
Response to stress	68	1337	1.77E-12	63	5	response to pest and pathogen, inflammatory response, response to virus, response to wounding
Inflammatory response	25	244	4.35E-11	25	0	defensive reaction (by vertebrate tissue) to infection or injury
Response to wounding	39	395	3.72E-16	39	0	defensive reaction (by vertebrate tissue) to physical injury
Signal transduction	123	3547	4.70E-06	95	17	cytokine signaling, death receptor signaling, G-protein coupled receptor signaling, intigrin binding, MHC protein binding
Receptor activity	72	1914	1.80E-07	62	10	chemokine receptors, pattern recognition receptors, immunoglobulin receptors, complement receptors, MHC I\MHCII receptors, scavenger receptors, Hematopoietin/interferon class (D200) receptors
Antigen binding	8	91	2.66E-03	8	0	*SCF1, IGLV2-14, LAG3, LILRA1, SLAMF1, TRA@, TRGC2, TAP2*
Carbohydrate binding	16	326	1.18E-03	14	2	*CCL8, CD69, CLEC4A, FCN1, FGFR2, GALNT7, HMMR, KLRC1/KLRC2, KLRF1, LGALS2, LGALS9, POSTN, PTN, SELL, SELPLG, SN*
Pattern binding	10	159	4.58E-02	8	2	*CCL8, CD14, FGFR2, HMMR, POSTN, PTN, TLR2, TLR7, TLR8, TLR4*
Cytokine receptors	9	99	1.97E-04	9	0	*CCR5, CCR1, CD74, CSF2RB, CXCR4, IL10RA, IL1RL1, IL21R, TNFRSF1B*
Cytokines	12	257	1.11E-04	11	1	*CCL3, CCL5, CCL8, CXCL5, CXCL10, CXCL11, CXCL12, CXCL16, TNFSF10, ECGF1, PTN, SLURP1*
Immunoglobulin binding	4	25	1.56E-03	4	0	*FCER1A, FCER1G, FCGR3B*, *214511_x_at*
Cystein type endopeptidase	9	169	8.21E-03	9	0	*CASP1, CASP8, CTSB, CTSL, CTSC, STSS, LGMN, TNFAIP3, USP18*

^1^Number of genes in the data for which annotation was found in the Gene Ontology database.

^2^Total number of gene with annotation in the Gene Ontology database.

^3^P-value for representation of gene ontology category in the data.

### Validation of selected genes observed in the Affymetrix experiment using real time reverse transcriptase polymerase chain reaction

**Figure 2:**
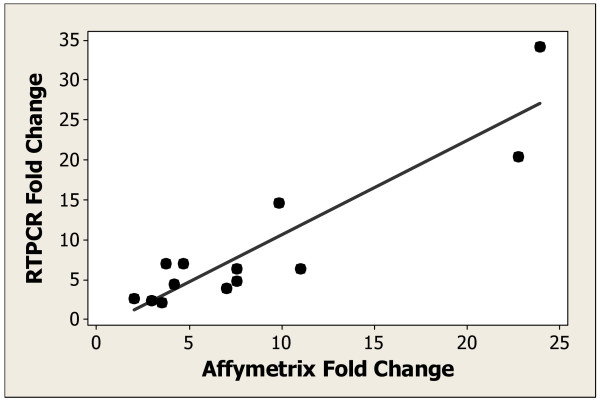
Comparison of gene expression data obtained by Affymetrix GeneChip analysis and real time RT-PCR. Linear regression analysis for *IRF7 *comparing fold change values for individual subjects as measured by Affymetrix analysis and real time RT-PCR analysis. The imiquimod response fold change, which is the maximum response from week 1, week 2 and week 4 treatment times was used. Fold change was calculated relative to sun-unexposed, nonlesional skin samples for 13 subjects treated with imiquimod. The R square value for the comparison was 0.83.

Micro array analysis may not be sensitive enough to capture all changes in gene expression. In comparison, real time reverse transcriptase polymerase chain reaction (RT-PCR) has been shown to be more sensitive for the detection of low abundance transcripts [31]. Therefore, we analyzed AK samples and imiquimod treated AK samples for a selected set of genes using real time RT-PCR. The data is summarized in [Additional File 2] which compares the median fold change values (13 imiquimod-treated subjects) measured using both Affymetrix GeneChip analysis and RT-PCR for a subset of the genes. In general, good agreement was obtained between the 2 methods, with the RT-PCR data showing similar or higher expression than the Affymetrix data. Figure 2 shows a comparison of the 2 methods for the expression of interferon regulatory factor 7 (IRF7). The individual fold change values for the 13 imiquimod-treated subjects during imiquimod treatment were used for the regression analysis (R Square = 0.83). In general, the direction of change in expression for each gene was the same in both assays whereas the magnitude of the fold change values was higher for the RT-PCR analysis than the Affymetrix analysis. Of the 46 genes evaluated on both gene expression platforms, only 3 genes (CD80, MyD88 and TLR6) were identified as having changed in expression in the RT-PCR experiments only (p value ANOVA analysis < 0.05). We did not identify any genes that were differentially expressed by the Affymetrix method that were not also differentially expressed by real time RT-PCR. Thus, overall good agreement was obtained between the 2 methods, with differences between the 2 methods likely due to the increased sensitivity and quantitative nature of the RT-PCR platform.

### Imiquimod increases expression of pattern-recognition receptors of the innate immune system

**Figure 3:**
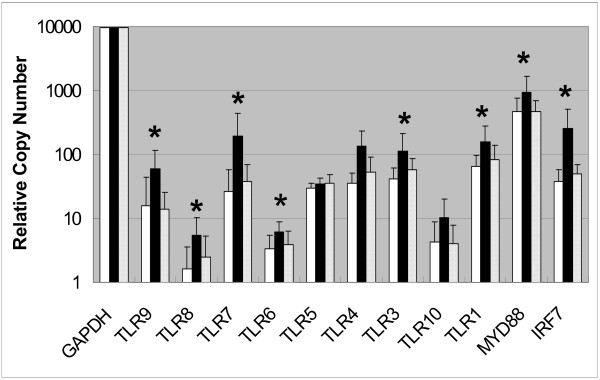
Basal *TLR*, *IRF7*, and *MyD88 *gene expression in skin biopsies as determined by real time RT-PCR. White bars represent pretreatment AK, black bars represent during imiquimod treatment (maximum response value from week 1, week 2 and week 4 treatment times), and hatched bars represent 4-weeks post treatment. Relative copy number was determined as outlined in Methods and Materials section. Asterisks indicate those genes that had p-values < 0.05 in the ANOVA, comparing expression in pretreatment AK samples to the maximum response expression in samples from subjects (n = 13) during imiquimod treatment. [See Additional file 2].

Since imiquimod is a TLR7 agonist, we sought to determine if *TLR7 *and other TLRs were expressed in AK lesions and if treatment with imiquimod altered their expression. We analyzed expression levels of various TLRs in biopsy samples of pretreatment AK and after treatment with imiquimod using RT-PCR. We also measured the expression levels of *MyD88*, an adaptor molecule for various TLRs including *TLR7*; and *IRF7*, a transcription factor recently shown to be important in the regulation of interferon gene expression through the TLR7 pathway [34]. Figure 3 shows the median expression levels of the various TLRs, *MyD88 *and *IRF7 *for subjects treated with imiquimod (n = 13) during treatment and 4 weeks post treatment. The expression of *TLR1*, *TLR3*, *TLR6*, *TLR7*, *TLR8*, *TLR9*, *MyD88 *and *IRF7 *were all increased at statistically significant levels (p-value < 0.05, during treatment [see also Additional File 2]. The increase in *TLR4 *expression did not reach a statistically significant level (p-value = 0.066) in the imiquimod-treated samples, whereas the Affymetrix data shows a statistically significant increase (p-value of 0.028). The most statistically significant changes observed upon treatment with imiquimod were for *TLR3*, *TLR7*, *TLR8*, and *IRF7*, with good agreement between the Affymetrix and RT-PCR analysis both for the magnitude of change and for the p-values. There was no change in the expression levels of *TLR5 *and *TLR10 *upon treatment with imiquimod. The data are consistent with previous reports of induction of TLRs by various TLR agonists and IFNα [35-37][38,39]. The increased expression of the TLRs may be a result of increased expression of the genes in cells resident in the skin (e.g., DCs, macrophages) or due to the influx of cells with high expression of these genes (e.g., DCs, macrophages, plasmacytoid DCs). The increased expression of *TLR3*, *TLR7*, and *TLR8 *is consistent with increased expression observed in human peripheral blood mononuclear cells upon treatment with imiquimod. The data are also consistent with increased expression of *TLR7 *observed in imiquimod-treated AK [22]. In summary, treatment of AK lesions with imiquimod results in increased expression of several TLRs and TLR pathway components, thus potentially priming for further amplification of the innate immune system. The increase in the expression of *IRF7 *is also predicted to amplify the innate immune response by increased expression of type 1 interferons and interferon-inducible genes.

**Figure 4:**
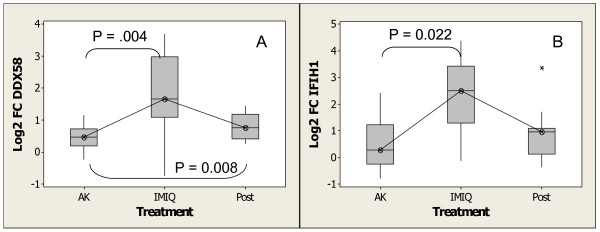
Increase in expression of the helicase family of virus-sensing genes upon treatment with imiquimod as determined by Affymetrix GeneChip analysis. (A)*DDX58 *(*RIG-I*) and (B)*IFIH1 *(*MDA5*). Experimental conditions are as described in the Methods and Materials section. Box plots were generated using MINITAB version 14. 'AK,' 'IMIQ,' and 'Post' designate pretreatment AK, imiquimod-treated skin during treatment (maximum response from week 1, week 2 and week 4), and imiquimod-treated skin 4 weeks post treatment, respectively. Fold change was calculated with respect to sun-unexposed skin. Boxes indicate the median 95% confidence intervals, asterisks designate outliers, and the lines connect median values. P values are given for 2-way ANOVA, comparing the fold change values for pretreatment AK samples and imiquimod-treated samples (n = 13).

In addition to the various TLR(s) which recognize viral and bacterial components, an intracellular antiviral pathway which detects viral RNA and results in the induction of type1 interferons has recently been described [40-42]. The central components of this pathway, which are also inducible by type1 interferons [43,44] are *DDX58 *(*RIG-I*, retinoid acid inducible gene) and *IFIH1 *(*MDA5*, melanoma differentiation antigen 5). Both genes contain helicase domains responsible for detection of double-stranded RNA. They also contain caspase recruitment domains (CARDdomains), which are responsible for signaling through *TBK1 *(resulting in activation of *NFKB *and *IRF3*), as well as induction of type-1 interferons. The activation of this pathway leads to growth inhibition as well as antiviral activity [45]. Figure 4a and 4b show changes in expression of *DDX58 *and *IFIH1 *upon treatment with imiquimod as determined from Affymetrix GeneChip analysis. The expression of both genes was increased to statistically significant levels during treatment. The expression of *DDX58 *remained higher than its pretreatment level (p-value = 0.004), four weeks, post treatment, whereas that of *IFIH1 *returned to basal level. The induction of several members of the cytoplasmic helicase innate immune pathway, as well as several TLRs, indicates that in addition to activation of the TLR7 pathway, treatment with imiquimod also results in priming of other innate pathways. These pathways may augment other aspects of the innate immune response and may be important for eliminating pre-neoplastic cells.

### Imiquimod induces a large number of type I interferon-inducible genes with growth inhibitory and immune-stimulatory activity

Imidazoquinoline TLR7 agonists such as imiquimod and resiquimod are know to induce various cytokines, including interferon-α, IL6, MCP-1 and IL12 as well as the co-stimulatory markers CD80 and CD86 [15,46]. Type I interferons are known to be powerful regulators of the innate and adaptive immune system through the induction of various genes with antiviral, anti-tumor and immune regulatory functions [43,44,47-51]. In this study, we did not detect increased expression of interferon upon treatment with imiquimod, but observed the increased expression of a large number of IFN-inducible genes (114 genes), [see Additional file 3]. The lack of detection of type 1 interferons after imiquimod treatment in this study may be due to the early induction and degradation of their respective mRNA. Biopsies were taken approximately 8 to 16 hr after application of the drug. We have observed that mRNA for type 1 interferons in human blood mononuclear cells treated with imiquimod peaks in 1 to 2 hours and declines to basal levels 6 to 8 hours post treatment (unpublished internal data).

**Figure 5:**
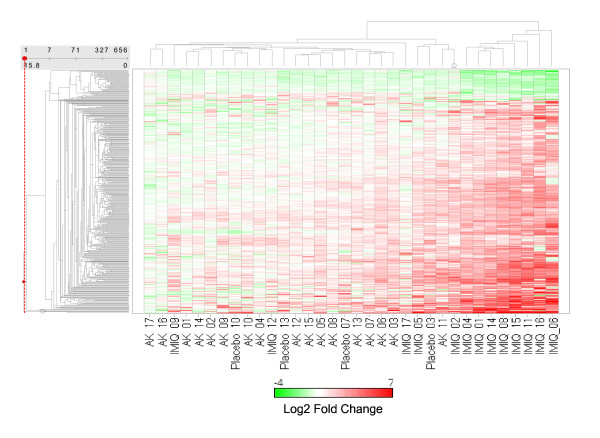
Cluster analysis of genes regulated by imiquimod treatment. Two-way hierarchical clustering was performed as described in the Methods and Materials section. 'AK,' 'IMIQ,' and 'Placebo' designate the fold change with respect to sun-unexposed, nonlesional skin for pretreatment AK samples; for samples during imiquimod treatment (maximum response from week 1, week 2 and week 4), and samples for vehicle-treatment (maximum response from week 1, week 2 and week 4) respectively. Numbers designate subjects. Hierarchical clustering was performed using the Unweighted Pair-Group Method with Arithmetic mean (UPGMA) and the Euclidean similarity measure. Red, white, and green indicate up-regulated, unchanged, and down-regulated genes, respectively. The color bar insert shows the corresponding expression levels. The cluster consists of 530 imiquimod-responsive genes whose expression was statistically different when comparing the AK group of samples to the IMIQ group of samples. Expression changes for the 530 genes are documented in [Additional file 1].

**Figure 6:**
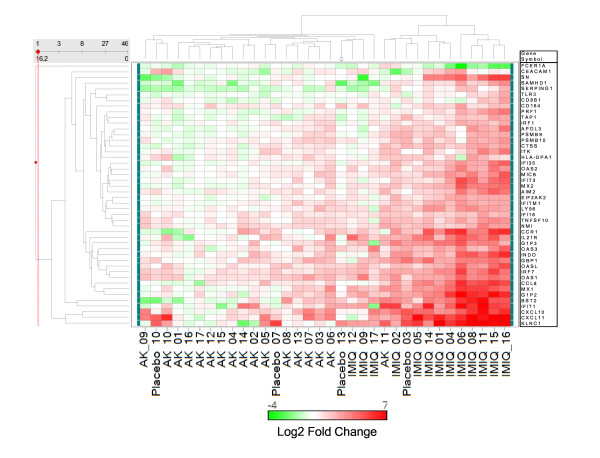
Cluster of 46 immune response genes that are also known to be inducible upon treatment with type 1 interferons as reported in [18, 43, 45, 48, 51, 102] Cluster analysis was performed as described in Figure 5. [See Additional file 3].

Analysis of gene ontology classification of the interferon-inducible genes increased by treatment with imiquimod identified 46 genes with immune response classification. Figure 5 shows a 2-way hierarchical clustering of the log2 transformed fold changes of all of the 530 imiquimod-induced genes [see Additional file 1]. Figure 6 exhibits clustering of the 46 interferon-inducible genes which classify as immune response genes Two main clusters are apparent in Figure 5. One cluster consists of 8 imiquimod-treated samples. The second and larger cluster consists of all of the pretreatment AK samples, the vehicle-treated samples (designated as placebo in the figure) and five of the imiquimod-treated samples for subjects 02, 05, 09, 12 and 17 (IMIQ-02, IMIQ-05, IMIQ-09, IMIQ-12 and IMIQ-17). The fact that the vehicle-treated samples cluster with pretreatment AK lesions indicates the lack of a significant vehicle-effect in the gene expression profile. In Figure 6, 10 of the imiquimod-treated samples appear in 1 cluster, with IMIQ-02 and IMIQ-05 now as part of the IMIQ-treated cluster. This cluster is characterized by high expression of the interferon-inducible genes such as *MX1*, *IFIT1 *and *IFIT3*. The cluster also contains the samples placebo-03 and AK-11, indicating that these AK lesions were already manifesting spontaneous immune response. The imiquimod-treated samples 09, 12, and 17 are clustered with the pretreatment AK samples in both figures, indicating that Subjects 09, 12, and 17 had little gene expression response to imiquimod. Thus, the 46 interferon-inducible immune response genes segregate the imiquimod-treated samples from pretreatment AK and placebo-treated samples better than the whole set of 530 genes. This group of genes may therefore be good predictors of gene expression response to imiquimod. Collectively, these genes have been reported as 'interferon-signature' genes induced by several IFNα subtypes and IFNβ in monocytes [51].

Heterogeneity of pretreatment AK lesions was not documented by clinical assessment in this study. However, the gene expression profiles shown in Figure 5 and Figure 6 indicate heterogeneity in lesions. In addition to AK 11 and Placebo 03 in Figure 6 which cluster with the imiquimod treatment group indicating some level of immune response in these samples, other AK lesions also show low levels of expression of interferon inducible genes. For example, AK 06, AK 03, and AK 07 show low levels of expression of several interferon-inducible genes whereas AK 16, AK 17, AK 12 and AK 15 show normal to slightly depressed levels (Figure 6).

Interferon-inducible genes regulate diverse cellular processes, such as cell growth and differentiation, cell death, and T-cell co stimulation, activation, and migration. These genes have been reported to possess antiviral [52-54], pro-apoptotic [55-57], and anti-proliferative activities [58,59]. The interferon-inducible genes which increased following imiquimod treatment include those known to be induced by viruses as well as those with known anti-viral activity. These include the 2'5'-oligoadenylate synthetases *OAS1*, *OAS2*, *OAS3 *and *OASL*, the genes encoding the interferon-inducible proteins with tetratricopepetide repeats, *IFIT1*, *IFIT2*, and *IFIT3*, *IFTM1*, and other interferon inducible genes such as *IFI35*, *IFI16*, *MX1*, *MX2*, *EIF2AK2 *(*PRKR*), *G1P2 *(*ISG15*), *G1P3*, *ISG20*, *RSAD2 *(*Cig5*), *CCL8 (MCP2*), *CXCL10 (IP10) *and *CXCL11 *(*ITAC*) [43,44,47,49-52,54,60,61]. Several of the interferon-inducible genes were also increased at statistically significant levels 4 weeks post treatment. These included: *IRF7*, *IFI44*, *IFIT2, IFIT3, IFITM1, IFI35, RSAD2 (Cig5), G1P2, MX1, OAS1*, and *OAS2 *[see Additional file 1].

**Figure 7:**
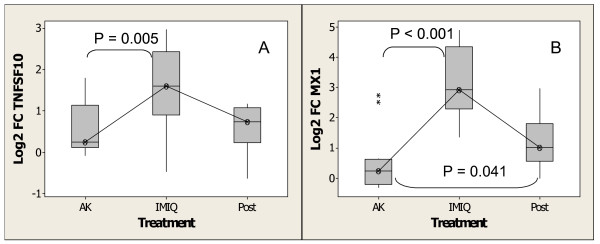
Imiquimod treatment is associated with an increase in expression of pro-apoptotic genes as determined by Affymetrix GeneChip analysis. (A) *TNFSF10 *(B)*MX1*. Experimental conditions and analysis are as described for Figure 4.

In addition to interferon-inducible genes with antiviral activity, several genes known or predicted to possess growth-inhibition and/or cell-differentiating activities [49,50] were induced by imiquimod, including *IFI16*, *AIM2 *[62,63], *IFIH1 *(melanoma differentiation antigen, *MDA5*) [45], *CXCL10 *(*IP10*) [58] and *EIF2AK2 (PRKR*) [64]. Some of the interferon-inducible genes also possess pro-apoptotic activity, including *MX1*, *TNFSF10 *(*TRAIL*), *OAS1*, and *PRF1 *[55,57,65]. Figure 7a and Figure 7b show changes in the expression of the pro-apoptotic genes *TNFSF10 *(*TRAIL*) and *MX1 *with imiquimod treatment as determined from the Affymetrix analysis. The expression of *MX1 *remained elevated at statistically significant level 4 weeks post treatment, whereas the expression of *TNFSF10 *returned to basal levels. The data are consistent with the observation of increased expression of IFNα-inducible genes in imiquimod-treated BCC and cutaneous T-cell lymphoma (CTCL) [66,67]. Thus, interferon-inducible genes with pro-apoptotic activity (e.g., *MX1, TNFSF10*) and growth inhibitory activity (e.g., *IFIH1, AIM2, IFI16*) may result in growth inhibition of neoplastic cells whereas those with immune-stimulatory activity such as *CXCL10 (IP10), CXCL11 *(*ITAC*), and *CCL8 *(*MCP2*) may facilitate cell-mediated lesion destruction by recruiting immune cells into the lesions. Indeed, further evidence for the recruitment of immune cells into AK lesions with imiquimod treatment is presented in the following sections.

### Imiquimod induces the expression of chemokines responsible for recruitment of immune cells to AK lesions

**Figure 8:**
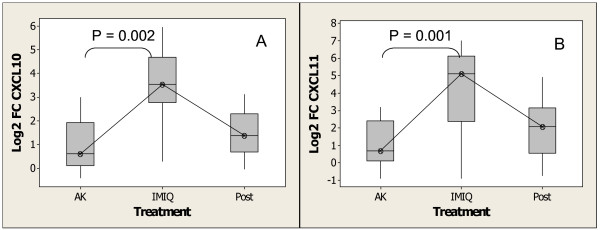
Increase in expression of chemokines after treatment with imiquimod as determined by Affymetrix GeneChip analysis. (A)*CXCL10 *(*IP10*), (B)*CXCL11 *(*ITAC*). Experimental conditions and analysis are as described for Figure 4.

Gene ontology classification (Table 1) shows that 106 genes classified as immune responsive were induced by imiquimod, some of which are also shown to be interferon α/β-inducible [see Additional File 3]. Out of the 106 immune response genes, several were classified as cytokines/chemokines and chemokine receptors. In addition to *CXCL10*, *CXCL11 *and *CCL8 *mentioned above, other chemokines were induced by imiquimod treatment, including *CCL3 *(*MIP1a*), *CCL4 *(*MIP1b*), *CCL5 *(Rantes), *CXCL12 *(*SDF1*), and *CXCL16*, and the chemokine receptors *CCR1*, *CCR5*, and *CXCR4*. Figure 8a and 8b illustrate changes in expression of *CXCL10 *and *CXCL11 *observed upon treatment of AK lesions with imiquimod. The magnitude of the changes observed in the expression of chemokine genes during treatment ranged from a median fold change value of 1.8 for *CXCL12 *(*SDF1*) to 40.8 for *CXCL11*. The expression of both genes returned to pretreatment levels 4 weeks post treatment. These data are consistent with previous reports of increased expression of chemokines genes and their receptors upon topical treatment of BCC and CTCL with imiquimod [67], as well as *in vitro *studies of human blood mononuclear cells stimulated with other imidazoquinoline TLR7 agonists showing the induction of CXCL10 and CXCL11 proteins [46]. The cocktail of chemokines up-regulated by imiquimod is consistent with recruitment and/or activation of macrophages, DCs, plasmacytoid DCs, gamma/delta T cells, cytotoxic T cells, and natural killer (NK) cells, and is also consistent with the cell surface markers indicating the presence of these cells upon imiquimod treatment. Indeed, topical treatment of various neoplastic skin conditions with imiquimod cream have shown inflammatory conditions at the site of treatment, indicating the infiltration of immune cells into the site [17,19,20,68,69]. In this study, the gene expression fingerprints that indicate recruitment of various immune cells are further discussed below.

### Imiquimod increases the expression of genes predictive of infiltrating macrophage and dendritic cells

Of the imiquimod-induced genes classified as immune response in gene ontology, several have receptor activity and (or) are hematopoietic cell surface markers (Table 1). The increased expression of these genes indicates the recruitment of various immune cell types to the lesion sites. Macrophage and/or monocyte infiltration of AK lesions upon treatment with imiquimod was indicated by an increase in *CD14*, *CD163*, and *CLECSF9 *(*CLECSF4*, *MINCLE*), a C-type lectin found on activated macrophages [70]. The increase in expression of genes of the classical complement pathway, *C1QA*, *C1QB*, *C3AR1*, and *C5R1*, also indicates an increase in and/or the activation of macrophages [71]. The data are consistent with histologic observation of macrophage and/or monocyte infiltration after application ofimiquimod in the treatment of AK [72] and in lentigo maligna [73,74].

**Figure 9:**
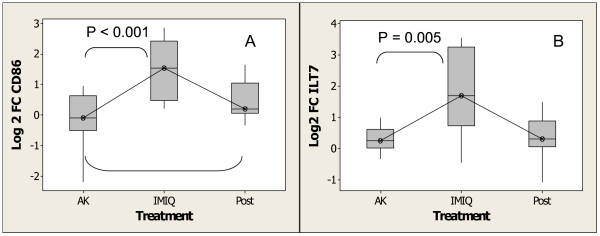
Increase in expression of genes indicating the infiltration of DCs upon treatment with imiquimod as determined by Affymetrix GeneChip analysis. (A)*CD86 *(B) *ILT7*. Experimental conditions and analysis are as described for Figure 4.

The presence of DCs is shown by increases in the co stimulatory molecules *CD86 *and *CLEC4A *(*DCIR*, *CLECSF6*), as well as by 3 leukocyte immunoglobulin receptors: *LILRB3 *(*ILT3*) and *LILRB1 *(*ILT2*) which are expressed in both myeloid and plasmacytoid DCs [75], while *ILT7 *(*LILRA4*, *CD85g*) is restricted to plasmacytoid DCs [76,77]. Figure 9a and Figure 9b illustrate the increase in expression of *CD86 *and ILT7 with imiquimod treatment. The expression of *CD86 *remained elevated 4 weeks post treatment. Increased expression is taken as indication of recruitment of these cells to the site of treatment. These observations are consistent with previous studies using topically-applied imiquimod in the treatment of human BCC [69] and melanoma in mice [78], showing recruitment of plasmacytoid DCs into the site of treatment.

It is interesting to note that *CD1C *is markedly decreased upon treatment with imiquimod [see Additional file 3]. CD1c is found on Langerhans cells as well as on DCs [79,80]. The decrease in *CD1C *expression may reflect (although not exclusively) the migration of CD1C+ Langerhans cells out of the dermis. Migration of Langerhans cells out of mouse dermis was observed after topical treatment with imiquimod [78,81]. In the case of the Palamara studies [78] in mouse melanoma, Langerhans cells were observed to return to normal levels in the dermis by day 20. The decrease in *CD1C *observed upon treatment of AK lesions with imiquimod therefore suggests the activation and migration of Langerhans cells to the lymph nodes and is consistent with previous observations.

### Imiquimod increases the expression of genes predictive of infiltrating cytotoxic T cells and natural killer cells

**Figure 10:**
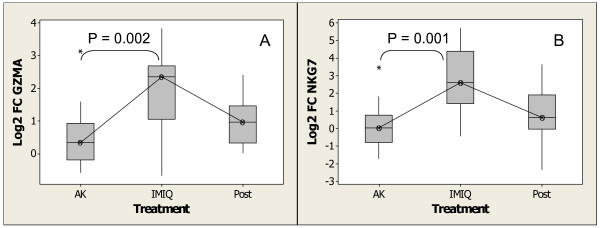
Increase in expression of genes associated with NK cells, and cytotoxic T cells after treatment with imiquimod as determined by Affymetrix gene expression. (A)*GZMA *(B) *NKG7*. Experimental conditions and analysis are as described for Figure 4.

Natural killer cells mediate lysis of tumor cells as well as virally-infected cells. Natural killer cells preferentially express several calcium-dependent (C-type) lectins, known as the NKG2 family, which have been implicated in the regulation of NK cell function, and are believed to be important for NK cell-mediated tumor rejection and T-cell mediated immunity [82]. Transcripts for 3 of these C-type lectins, *KLRC1*/*C2*, *KLRK1 *(*NKG2D*) and *KLRF1 *were increased in expression in imiquimod-treated samples. In addition, several genes important to the cytolytic function of NK cells and cytotoxic T cells, including *TYROBP *(*DAP12*) and *CD69 *(early T-cell activation antigen), *ITGAL *(*CD11a*), *ITGB2 *(*CD18*), *ICAM1*, and the ligands for the *NKG2 *receptors, *MICA *and *MICB *[Additional file 1] [83-88] were increased in expression with imiquimod treatment. Also, genes of the granule products of NK cells and cytotoxic T cells, such as granzymes *GZMA*, *GZMB*, *GZMK*, and *GYNYL *(granulysin, *NKG5*), *PRF *(perforin) and *NKG7 *(*GIG1*, *GMP-17*) were increased in expression, indicating that these cells are cytolytically active [89-93]. Figure 10a and 10b show changes in the expression of *GZMA *and *NKG7 *after imiquimod treatment. The expression of both genes returned to pretreatment levels 4 weeks post treatment. Thus, the coordinate increased expression of genes important for NK cell recognition of tumor cells, and for the activation and cytolytic response of NK cells and cytotoxic T cells, suggests that these cells are at least in part responsible for the antilesional activity of imiquimod.

### Imiquimod increases the expression of genes predictive of the activation of the adaptive immune system

**Figure 11:**
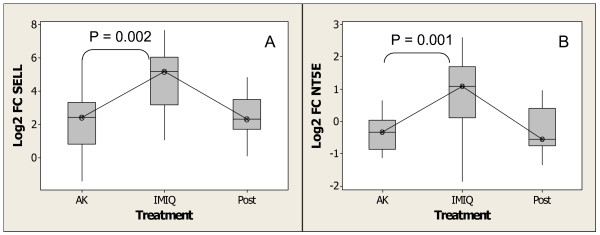
Increase in expression of genes associated with lymphocyte function after treatment with imiquimod as determined by Affymetrix GeneChip analysis. (A) *SELL *(*CD62L*) (B) *NT5E *(*DC73*). Experimental conditions and analysis are as described for Figure 4.

The presence of activated T cells in imiquimod-treated AK lesions is indicated by the expression of several members of the T-cell activation pathway [Additional File 1]). These include the T-cell receptor (TCR) subunits TRD and TRG, TCR-signaling pathway genes such as *Fyn*, *Fyb *and *LCP2 *[94,95], and other genes associated with T-cell activation such as *HCK*, *CD69*, *PTPRC *(*CD45*) *SELL *(*CD62L*, L-Selectin), *ITGA4 *(antigen CD49D, alpha 4 subunit of VLA-4 receptor), and *LAG3*. In addition, treatment of AK lesions with imiquimod resulted in a small but significant increase in the expression of CD8β, suggesting that there is an increase in CD8 T cells [96,97]. The presence of CD8 memory T cells in imiquimod-treated subjects is also suggested by increases in expression of *SELL*, *NT5E *(*CD73*), *LGALS2 *(galectin 2), and *LAIR1 *(leukocyte-associated immunoglobulin-like receptor 1), which was recently identified to be differentially expressed in mouse memory Tcells [98]. Figure 11a and Figure 11b illustrate the increase in expression of *SELL *and NT5E respectively. The expression levels of these genes returned to pretreatment levels 4 weeks post treatment. Taken together, the increased expression of Tcell receptor genes, T cell activation marker genes and genes important for T cell co stimulation suggests that treatment with imiquimod results in the infiltration of T cells into AK lesions. The increased expression of genes important for the development of T-cell memory (e.g. *SELL*, *NT5E*), as well as genes associated with cytotoxic T cells (e.g. granzymes and perforin) suggests that imiquimod treatment recruits both memory and effector T cells. These results are consistent with the observation of increased CD3, CD4 and CD8 positive cells after topical application of imiquimod to AK lesions [72], as well as infiltration of CD8 T cells in BCC [19,66] and in cutaneous squamous cell carcinoma treated with topical imiquimod [99]. The increased expression of genes important in the activation and co stimulation of cells of the adaptive immune system is consistent with the infiltration of cells important in the development of the adaptive immune system. These observations are also consistent with the reported adjuvant activity of imiquimod and its analog resiquimod [26], and the low recurrence rates observed in animal studies [100] and clinical studies with imiquimod [101]. These same observations suggest that imiquimod may also prevent recurrence of AK lesions as well.

In summary, the data suggest that the therapeutic effect of imiquimod in the treatment of AK involves the stimulation of both the innate and adaptive immune responses. The role of type 1 interferons in imiquimod's mechanism of action is underscored by the induction of large numbers of IFNα/β-inducible genes with tumor growth-inhibitory and immune stimulatory activity. Data indicate that the antilesional activity of imiquimod is a result of the induction of a strong immune cell-mediated cytolytic and apoptotic gene expression program that leads to destruction of AK lesions and sun-damaged cells. The development of immune memory indicated by this study, as well as the observation of low recurrence in clinical studies, makes imiquimod a unique therapy for AK as well as for other cutaneous neoplasms.

## Abbreviations

AK–actinic keratosis(ses), ANOVA–analysis of variance, BCC–basal cell carcinoma, DC–dendritic cells, IFN–interferon, IL–interleukin, IRF–interferon regulatory factor, NK–natural killer [cells], RT-PCR–Reverse Transcriptase Polymerase Chain Reaction, TLR–Toll like receptors

## Competing interests

Abel Torres, MD, JD, has acted as a lecturer for 3M Pharmaceutical and has participated in 3M Pharmaceutical funded research. He also does consulting for 3M Pharmaceuticals.

RM, BJB, JJ, SR, and WB are employees of 3M Pharmaceuticals. JL and HB were employees of 3M Pharmaceuticals at the time of the study and manuscript writing.

## Authors' contributions

AT, LS and MA conducted the clinical study. JL was the clinical coordinator. Gene expression analysis was performed at 3M Pharmaceuticals by WB, BB and JJ. All authors approved the manuscript.

## Supplementary Material

Additional file 1 Imiquimod regulated genes. This file summarizes fold change in expression for 530 imiquimod regulated genes before and after treatment with imiquimod. Genes were selected on the basis of an ANOVA analysis comparing pre-treatment AK expression values to expression values during imiquimod treatment or 4-weeks after treatment with imiquimod, with P-values < 0.05 Click here for file

Additional file 2 Comparison of differential expression as measured by Affymetrix gene array and real time RT-PCR. This file summarizes a comparison of differential gene expression as measured by Affymetrix gene chip analysis and real time RT-PCR using low density TaqMan arrays. Expression changes for several toll-like receptors and other selected genes before and after treatment with imiquimod are reported. Click here for file

Additional file 3 Imiquimod-induced genes with immune response gene ontology category and/or known to be IFNα/β-inducible. This file reports on expression changes before and after treatment with imiquimod, for selected genes with gene ontology category of immune response and (or) are know to be inducible by type 1 interferons. Click here for file
