# Supplementary material for: Immune-mediated changes in actinic keratosis following topical treatment with imiquimod 5% cream
Source: J Transl Med. 2007 Jan 26;5:7. doi: 10.1186/1479-5876-5-7 (PMC1796543; doi:10.1186/1479-5876-5-7)
Supplement: Additional file 1 — Imiquimod regulated genes. This file summarizes fold change in expression for 530 imiquimod regulated genes before and after treatment with imiquimod. Genes were selected on the basis of an ANOVA analysis comparing pre-treatment AK expression values to expression values during imiquimod treatment or 4-weeks after treatment with imiquimod, with P-values < 0.05 [file 1479-5876-5-7-S1.doc]

| **Additional file 1. Imiquimod regulated genes** | | | | | | | |
| --- | --- | --- | --- | --- | --- | --- | --- |
| Affymetrix Probe Set ID | Gene Symbol | FC^1^ AK | FC^1^ IMIQ | P-value^2^ IMIQ Vs AK | FC^1^ 4wk Post | P-value^3^ 4WK Post Vs AK | Gene Title |
| 227353_at | --- | 1.4 | 2.4 | 0.011 | 1.60 | **0.004** | --- |
| 229121_at | --- | 1.0 | 2.3 | 0.000 | 1.29 | **0.027** | CDNA FLJ44441 fis, clone UTERU2020242 |
| 243366_s_at | --- | 1.0 | 3.7 | 0.046 | 2.25 | **0.044** | Transcribed locus |
| 236401_at | --- | -1.1 | 2.3 | 0.012 | 1.05 | 0.059 | --- |
| 230866_at | --- | -1.1 | 1.9 | 0.006 | 1.29 | 0.064 | --- |
| 233853_at | --- | 1.3 | -1.1 | 0.001 | 1.21 | 0.065 | Similar to RAB guanine nucleotide exchange factor (GEF) 1 |
| 213929_at | --- | -1.8 | -3.2 | 0.035 | -2.01 | 0.071 | Homo sapiens, Similar to likely ortholog of yeast ARV1, clone IMAGE:4733238, mRNA |
| 201422_at | --- | 1.3 | 3.0 | 0.000 | 1.59 | 0.089 | --- |
| 215014_at | --- | -1.4 | -2.3 | 0.008 | -1.39 | 0.127 | MRNA; cDNA DKFZp547P042 (from clone DKFZp547P042) |
| 239237_at | --- | 1.3 | 2.7 | 0.030 | 1.62 | 0.128 | LOC442534 |
| 220146_at | --- | 1.4 | 4.9 | 0.000 | 1.51 | 0.140 | --- |
| 242521_at | --- | 1.8 | 3.7 | 0.001 | 1.84 | 0.146 | CDNA clone IMAGE:30349460, partial cds |
| 237753_at | --- | 1.5 | 2.3 | 0.038 | 1.64 | 0.199 | Transcribed locus, moderately similar to XP_522527.1 similar to carnitine deficiency-associated gene expressed in ventricle 1 [Pan troglodytes] |
| 227167_s_at | --- | 1.1 | 1.4 | 0.042 | 1.24 | 0.203 | Mesenchymal stem cell protein DSC96 |
| 226725_at | --- | 1.3 | 2.3 | 0.005 | 1.85 | 0.258 | Transcribed locus |
| 214511_x_at | --- | 2.0 | 12.4 | 0.007 | 2.22 | 0.375 | --- |
| 213675_at | --- | -1.4 | -2.5 | 0.004 | -1.30 | 0.408 | CDNA FLJ25106 fis, clone CBR01467 |
| 243955_at | --- | 1.9 | 1.2 | 0.018 | 1.91 | 0.439 | Transcribed locus |
| 227384_s_at | --- | 1.2 | 1.7 | 0.037 | 1.26 | 0.612 | Similar to KIAA0454 protein |
| 237530_at | --- | 1.9 | -1.3 | 0.032 | 1.78 | 0.648 | Transcribed locus |
| 205857_at | --- | -1.4 | -2.1 | 0.045 | -1.26 | 0.662 | --- |
| 227361_at | --- | 1.9 | 3.4 | 0.003 | 1.40 | 0.708 | --- |
| 228826_at | --- | 1.3 | 1.8 | 0.031 | 1.28 | 0.819 | Homo sapiens, clone IMAGE:5215917, mRNA |
| 239231_at | --- | 1.3 | 2.6 | 0.047 | 1.30 | 0.828 | CDNA FLJ41910 fis, clone PEBLM2007834 |
| 227356_at | --- | 1.4 | 2.0 | 0.013 | 1.28 | 0.874 | CDNA: FLJ22198 fis, clone HRC01218 |
| 238466_at | --- | 2.0 | 1.1 | 0.031 | 1.53 | 0.952 | CDNA FLJ33443 fis, clone BRALZ1000103 |
| 226773_at | --- | 1.4 | 1.8 | 0.041 | 1.44 | 0.968 | MRNA (clone ICRFp507I1077) |
| 221641_s_at | ACATE2 | -1.1 | 1.6 | 0.007 | -1.05 | 0.493 | likely ortholog of mouse acyl-Coenzyme A thioesterase 2, mitochondrial |
| 202767_at | ACP2 | -1.0 | 1.6 | 0.014 | 1.26 | **0.001** | acid phosphatase 2, lysosomal |
| 215051_x_at | AIF1 | 1.2 | 4.9 | 0.022 | 1.65 | 0.155 | allograft inflammatory factor 1 |
| 206513_at | AIM2 | 1.5 | 2.9 | 0.002 | 1.29 | 0.443 | absent in melanoma 2 |
| 202760_s_at | AKAP2 | -1.3 | 1.7 | 0.018 | -1.13 | 0.477 | A kinase (PRKA) anchor protein 2 /// PALM2-AKAP2 protein |
| 225701_at | AKNA | 1.1 | 2.9 | 0.004 | 1.23 | 0.466 | AT-hook transcription factor |
| 201425_at | ALDH2 | -1.2 | -1.5 | 0.026 | -1.17 | 0.174 | aldehyde dehydrogenase 2 family (mitochondrial) |
| 221589_s_at | ALDH6A1 | -1.5 | -1.9 | 0.030 | -1.16 | 0.113 | Aldehyde dehydrogenase 6 family, member A1 |
| 228094_at | AMICA1 | 1.7 | 3.1 | 0.049 | 2.07 | 0.276 | adhesion molecule, interacts with CXADR antigen 1 |
| 227607_at | AMSH-LP | 1.4 | 2.5 | 0.033 | 1.35 | 0.365 | associated molecule with the SH3 domain of STAM (AMSH) like protein |
| 230925_at | APBB1IP | 1.1 | 4.2 | 0.008 | 1.66 | 0.212 | amyloid beta (A4) precursor protein-binding, family B, member 1 interacting protein |
| 206632_s_at | APOBEC3B | 1.8 | 2.6 | 0.014 | 1.83 | 0.768 | apolipoprotein B mRNA editing enzyme, catalytic polypeptide-like 3B |
| 204205_at | APOBEC3G | 1.1 | 3.9 | 0.007 | 1.68 | 0.120 | apolipoprotein B mRNA editing enzyme, catalytic polypeptide-like 3G |
| 221653_x_at | APOL2 | 1.8 | 4.7 | 0.004 | 2.06 | 0.151 | apolipoprotein L, 2 |
| 221087_s_at | APOL3 | 1.0 | 2.1 | 0.011 | 1.15 | 0.282 | apolipoprotein L, 3 |
| 219716_at | APOL6 | 1.6 | 5.4 | 0.003 | 2.08 | 0.190 | apolipoprotein L, 6 |
| 206177_s_at | ARG1 | 1.8 | -1.3 | 0.039 | 1.31 | 0.122 | arginase, liver |
| 218870_at | ARHGAP15 | 1.5 | 2.6 | 0.002 | 1.49 | 0.201 | Rho GTPase activating protein 15 |
| 204882_at | ARHGAP25 | 1.4 | 4.8 | 0.004 | 1.96 | 0.239 | Rho GTPase activating protein 25 |
| 226906_s_at | ARHGAP9 | 1.6 | 3.6 | 0.008 | 1.55 | 0.928 | Rho GTPase activating protein 9 |
| 201954_at | ARPC1B | 1.6 | 2.8 | 0.028 | 1.75 | 0.376 | actin related protein 2/3 complex, subunit 1B, 41kDa |
| 204244_s_at | ASK | 1.4 | 1.9 | 0.036 | 1.31 | 0.359 | activator of S phase kinase |
| 219918_s_at | ASPM | 1.9 | 2.0 | 0.022 | 1.74 | 0.831 | asp (abnormal spindle)-like, microcephaly associated (Drosophila) |
| 204998_s_at | ATF5 | 1.1 | 1.7 | 0.001 | 1.14 | 0.083 | activating transcription factor 5 |
| 205197_s_at | ATP7A | -1.2 | -1.7 | 0.040 | -1.45 | 0.193 | ATPase, Cu++ transporting, alpha polypeptide (Menkes syndrome) |
| 205363_at | BBOX1 | -1.3 | -2.0 | 0.029 | -1.39 | 0.064 | butyrobetaine (gamma), 2-oxoglutarate dioxygenase (gamma-butyrobetaine hydroxylase) 1 |
| 205681_at | BCL2A1 | 1.9 | 5.4 | 0.047 | 1.85 | 0.842 | BCL2-related protein A1 |
| 211725_s_at | BID | 1.6 | 2.3 | 0.043 | 1.58 | 0.615 | BH3 interacting domain death agonist /// BH3 interacting domain death agonist |
| 204860_s_at | BIRC1 | 1.3 | 3.7 | 0.003 | 1.48 | **0.039** | baculoviral IAP repeat-containing 1 |
| 210538_s_at | BIRC3 | 1.9 | 3.8 | 0.012 | 1.84 | 0.615 | baculoviral IAP repeat-containing 3 |
| 201641_at | BST2 | -1.1 | 11.2 | 0.000 | 2.67 | 0.285 | bone marrow stromal cell antigen 2 |
| 212613_at | BTN3A2 | 1.1 | 2.9 | 0.005 | 1.34 | 0.200 | butyrophilin, subfamily 3, member A2 |
| 204821_at | BTN3A3 | -1.1 | 2.5 | 0.016 | 1.44 | **0.028** | butyrophilin, subfamily 3, member A3 |
| 225929_s_at | C17orf27 | -1.1 | 2.7 | 0.000 | 1.65 | **0.010** | chromosome 17 open reading frame 27 |
| 207571_x_at | C1orf38 | 1.2 | 3.1 | 0.009 | 1.62 | 0.457 | chromosome 1 open reading frame 38 |
| 218232_at | C1QA | 1.0 | 2.8 | 0.000 | 1.84 | **0.003** | complement component 1, q subcomponent, alpha polypeptide |
| 202953_at | C1QB | 1.1 | 4.1 | 0.001 | 2.03 | 0.051 | complement component 1, q subcomponent, beta polypeptide |
| 232150_at | C20orf18 | -1.4 | 1.4 | 0.027 | 1.10 | **0.006** | Chromosome 20 open reading frame 18 |
| 209906_at | C3AR1 | 1.0 | 2.6 | 0.024 | 2.07 | 0.108 | complement component 3a receptor 1 |
| 220088_at | C5R1 | 1.1 | 5.1 | 0.009 | 1.66 | 0.583 | complement component 5 receptor 1 (C5a ligand) |
| 209829_at | C6orf32 | 1.3 | 2.9 | 0.010 | 1.72 | 0.388 | chromosome 6 open reading frame 32 |
| 226603_at | C7orf6, SAMD9L | 1.2 | 4.2 | 0.000 | 2.01 | **0.015** | chromosome 7 open reading frame 6 |
| 225604_s_at | C9orf19 | 1.1 | 1.9 | 0.001 | 1.33 | 0.241 | chromosome 9 open reading frame 19 |
| 218904_s_at | C9orf40 | 1.1 | 1.4 | 0.013 | 1.01 | 0.834 | chromosome 9 open reading frame 40 |
| 218979_at | C9orf76 | 1.1 | 1.3 | 0.022 | 1.00 | 0.706 | chromosome 9 open reading frame 76 |
| 218384_at | CARHSP1 | 2.0 | 2.6 | 0.036 | 1.56 | 0.436 | calcium regulated heat stable protein 1, 24kDa |
| 211368_s_at | CASP1 | 1.4 | 2.1 | 0.009 | 1.40 | 0.881 | caspase 1, apoptosis-related cysteine protease (interleukin 1, beta, convertase) |
| 213373_s_at | CASP8 | 1.1 | 1.9 | 0.002 | 1.43 | 0.245 | caspase 8, apoptosis-related cysteine protease |
| 201432_at | CAT | -1.5 | -2.0 | 0.034 | -1.43 | 0.830 | catalase |
| 201091_s_at | CBX3 | 1.6 | 2.4 | 0.000 | 1.75 | 0.693 | chromobox homolog 3 (HP1 gamma homolog, Drosophila) |
| 225297_at | CCDC5 | 1.3 | 1.8 | 0.031 | 1.37 | 0.874 | coiled-coil domain containing 5 (spindle associated) |
| 205114_s_at | CCL3 /// CCL3L1 /// MGC12815 | 1.8 | 6.1 | 0.006 | 1.61 | 0.802 | chemokine (C-C motif) ligand 3 /// chemokine (C-C motif) ligand 3-like 1 /// chemokine (C-C motif) ligand 3-like, centromeric |
| 1405_i_at | CCL5 | 1.1 | 19.4 | 0.001 | 3.18 | **0.020** | chemokine (C-C motif) ligand 5 |
| 214038_at | CCL8 | 1.5 | 10.7 | 0.000 | 1.55 | 0.111 | chemokine (C-C motif) ligand 8 |
| 214710_s_at | CCNB1 | 1.8 | 2.6 | 0.010 | 1.75 | 0.736 | cyclin B1 |
| 205034_at | CCNE2 | 1.3 | 1.9 | 0.001 | 1.49 | 0.611 | cyclin E2 |
| 205099_s_at | CCR1 | 1.2 | 9.3 | 0.000 | 2.28 | 0.160 | chemokine (C-C motif) receptor 1 |
| 206991_s_at | CCR5 | 2.0 | 5.0 | 0.005 | 2.55 | 0.244 | chemokine (C-C motif) receptor 5 |
| 229900_at | CD109 | 1.4 | 1.8 | 0.012 | 1.48 | 0.109 | CD109 antigen (Gov platelet alloantigens) |
| 201743_at | CD14 | 1.1 | 4.0 | 0.013 | 1.34 | 0.132 | CD14 antigen /// CD14 antigen |
| 215049_x_at | CD163 | -1.0 | 5.0 | 0.000 | 1.37 | 0.122 | CD163 antigen |
| 208653_s_at | CD164 | 1.4 | 2.2 | 0.025 | 1.60 | 0.604 | CD164 antigen, sialomucin |
| 205987_at | CD1C | 1.1 | -6.1 | 0.005 | 1.38 | 0.162 | CD1C antigen, c polypeptide |
| 205831_at | CD2 | 1.6 | 5.0 | 0.019 | 2.50 | 0.113 | CD2 antigen (p50), sheep red blood cell receptor /// CD2 antigen (p50), sheep red blood cell receptor |
| 209933_s_at | CD300A | 1.1 | 1.9 | 0.001 | 1.06 | 0.644 | CD300A antigen |
| 205692_s_at | CD38 | 1.3 | 7.5 | 0.000 | 2.20 | 0.088 | CD38 antigen (p45) |
| 213857_s_at | CD47 | 1.8 | 2.6 | 0.041 | 1.84 | 0.844 | CD47 antigen (Rh-related antigen, integrin-associated signal transducer) |
| 237759_at | CD48 | 1.2 | 4.1 | 0.010 | 1.27 | 0.399 | CD48 antigen (B-cell membrane protein) |
| 203416_at | CD53 | 1.6 | 5.6 | 0.001 | 1.99 | 0.241 | CD53 antigen |
| 209795_at | CD69 | 1.8 | 6.3 | 0.046 | 1.73 | 0.690 | CD69 antigen (p60, early T-cell activation antigen) |
| 209619_at | CD74 | 1.1 | 1.6 | 0.048 | 1.21 | 0.082 | CD74 antigen (invariant polypeptide of major histocompatibility complex, class II antigen-associated) |
| 205685_at | CD86 | 1.1 | 2.9 | 0.000 | 1.53 | **0.020** | CD86 antigen (CD28 antigen ligand 2, B7-2 antigen) |
| 207979_s_at | CD8B1 | 1.0 | 1.4 | 0.018 | 1.19 | 0.457 | CD8 antigen, beta polypeptide 1 (p37) |
| 202910_s_at | CD97 | -1.3 | 1.3 | 0.005 | -1.09 | 0.781 | CD97 antigen |
| 202870_s_at | CDC20 | 1.4 | 1.8 | 0.042 | 1.65 | 0.183 | CDC20 cell division cycle 20 homolog (S. cerevisiae) |
| 209644_x_at | CDKN2A | 1.8 | 2.4 | 0.068 | 2.28 | **0.028** | cyclin-dependent kinase inhibitor 2A (melanoma, p16, inhibits CDK4) |
| 205709_s_at | CDS1 | -1.1 | -1.5 | 0.011 | -1.13 | 0.317 | CDP-diacylglycerol synthase (phosphatidate cytidylyltransferase) 1 |
| 206192_at | CDSN | -1.1 | -1.8 | 0.009 | -1.10 | 0.620 | corneodesmosin |
| 211889_x_at | CEACAM1 | -1.2 | 1.5 | 0.001 | 1.54 | **0.009** | carcinoembryonic antigen-related cell adhesion molecule 1 (biliary glycoprotein) |
| 203757_s_at | CEACAM6 | -1.2 | 1.8 | 0.021 | -1.13 | 0.758 | carcinoembryonic antigen-related cell adhesion molecule 6 (non-specific cross reacting antigen) |
| 219505_at | CECR1 | 1.5 | 3.8 | 0.012 | 2.14 | **0.020** | cat eye syndrome chromosome region, candidate 1 |
| 205250_s_at | Cep290 | 1.2 | 1.6 | 0.004 | 1.31 | 0.696 | centrosome protein cep290 |
| 204260_at | CHGB | -1.3 | 1.4 | 0.006 | 1.15 | **0.040** | chromogranin B (secretogranin 1) |
| 219947_at | CLEC4A | 1.5 | 2.3 | 0.008 | 1.71 | 0.072 | C-type lectin domain family 4, member A |
| 222934_s_at | CLEC4E | 1.0 | 2.9 | 0.032 | 1.65 | 0.098 | C-type lectin domain family 4, member E |
| 211964_at | COL4A2 | 1.3 | 2.0 | 0.044 | 1.59 | **0.040** | collagen, type IV, alpha 2 |
| 220356_at | CORIN | 1.8 | -1.7 | 0.070 | 2.53 | **0.035** | corin, serine protease |
| 207630_s_at | CREM | 1.1 | 1.7 | 0.036 | 1.24 | 0.888 | cAMP responsive element modulator |
| 206914_at | CRTAM | 1.7 | 2.6 | 0.016 | 1.85 | **0.020** | class-I MHC-restricted T cell associated molecule |
| 205159_at | CSF2RB | 1.3 | 4.1 | 0.007 | 1.68 | 0.759 | colony stimulating factor 2 receptor, beta, low-affinity (granulocyte-macrophage) /// colony stimulating factor 2 receptor, beta, low-affinity (granulocyte-macrophage) |
| 210140_at | CST7 | 1.2 | 2.5 | 0.029 | 1.64 | 0.066 | cystatin F (leukocystatin) |
| 215930_s_at | CTAGE5 | 1.5 | 1.9 | 0.043 | 1.30 | 0.813 | CTAGE family, member 5 |
| 213274_s_at | CTSB | 1.4 | 2.2 | 0.005 | 1.58 | **0.041** | cathepsin B |
| 201487_at | CTSC | 1.5 | 2.6 | 0.039 | 1.62 | 0.727 | cathepsin C |
| 202087_s_at | CTSL | 1.2 | 4.7 | 0.001 | 1.47 | 0.083 | cathepsin L |
| 202902_s_at | CTSS | 1.2 | 3.2 | 0.001 | 1.56 | 0.122 | cathepsin S |
| 204533_at | CXCL10 | 1.5 | 12.6 | 0.002 | 2.60 | 0.253 | chemokine (C-X-C motif) ligand 10 |
| 210163_at | CXCL11 | 1.8 | 40.8 | 0.001 | 4.20 | 0.171 | chemokine (C-X-C motif) ligand 11 |
| 203666_at | CXCL12 | 1.4 | 1.8 | 0.014 | 1.64 | **0.003** | chemokine (C-X-C motif) ligand 12 (stromal cell-derived factor 1) |
| 223454_at | CXCL16 | 1.2 | 1.7 | 0.009 | 1.41 | 0.777 | chemokine (C-X-C motif) ligand 16 |
| 214974_x_at | CXCL5 | 1.4 | 3.4 | 0.023 | 1.23 | 0.934 | chemokine (C-X-C motif) ligand 5 |
| 209201_x_at | CXCR4 | 1.8 | 4.3 | 0.002 | 1.67 | 0.746 | chemokine (C-X-C motif) receptor 4 |
| 228752_at | CXorf10 | -1.1 | 1.4 | 0.032 | 1.16 | 0.344 | chromosome X open reading frame 10 |
| 204923_at | CXorf9 | 1.2 | 1.9 | 0.008 | 1.42 | 0.101 | chromosome X open reading frame 9 |
| 203028_s_at | CYBA | 1.4 | 3.4 | 0.009 | 1.82 | **0.005** | cytochrome b-245, alpha polypeptide |
| 203923_s_at | CYBB | 1.2 | 2.6 | 0.001 | 1.27 | 0.227 | cytochrome b-245, beta polypeptide (chronic granulomatous disease) |
| 205765_at | CYP3A5 | -1.8 | -14.7 | 0.004 | -2.51 | 0.386 | cytochrome P450, family 3, subfamily A, polypeptide 5 |
| 209570_s_at | D4S234E | -1.5 | -2.3 | 0.027 | -1.49 | 0.649 | DNA segment on chromosome 4 (unique) 234 expressed sequence |
| 218943_s_at | DDX58 | 1.4 | 4.1 | 0.004 | 1.69 | **0.008** | DEAD (Asp-Glu-Ala-Asp) box polypeptide 58 |
| 207431_s_at | DEGS1 | -1.7 | -3.7 | 0.015 | -1.66 | 0.296 | degenerative spermatocyte homolog 1, lipid desaturase (Drosophila) |
| 200862_at | DHCR24 | 1.2 | -1.2 | 0.025 | -1.02 | 0.166 | 24-dehydrocholesterol reductase |
| 201790_s_at | DHCR7 | 1.6 | 1.2 | 0.014 | 1.37 | 0.679 | 7-dehydrocholesterol reductase |
| 204602_at | DKK1 | -1.1 | 1.3 | 0.022 | 1.05 | 0.089 | dickkopf homolog 1 (Xenopus laevis) |
| 215629_s_at | DLEU2 /// BCMSUNL | 1.2 | 1.6 | 0.008 | 1.26 | **0.043** | deleted in lymphocytic leukemia, 2 /// BCMS upstream neighbor-like |
| 203764_at | DLG7 | 2.0 | 3.3 | 0.005 | 2.00 | 0.952 | discs, large homolog 7 (Drosophila) |
| 212820_at | DMXL2 | 1.3 | 3.5 | 0.000 | 1.52 | 0.247 | Dmx-like 2 |
| 232843_s_at | DOCK8 | 1.5 | 2.7 | 0.011 | 1.49 | 0.320 | dedicator of cytokinesis 8 |
| 223553_s_at | DOK3 | 1.3 | 2.1 | 0.036 | 1.39 | 0.164 | docking protein 3 |
| 213071_at | DPT | -1.6 | -5.6 | 0.008 | -1.52 | 0.108 | dermatopontin |
| 207324_s_at | DSC1 | -1.1 | -2.3 | 0.013 | -1.39 | 0.191 | desmocollin 1 |
| 205419_at | EBI2 | 1.5 | 4.3 | 0.007 | 1.53 | 0.755 | Epstein-Barr virus induced gene 2 (lymphocyte-specific G protein-coupled receptor) |
| 217497_at | ECGF1 | 1.6 | 2.9 | 0.033 | 1.80 | 0.822 | endothelial cell growth factor 1 (platelet-derived) |
| 219454_at | EGFL6 | 1.6 | 4.3 | 0.001 | 2.27 | 0.104 | EGF-like-domain, multiple 6 |
| 204211_x_at | EIF2AK2 | 1.1 | 3.8 | 0.000 | 1.29 | 0.129 | eukaryotic translation initiation factor 2-alpha kinase 2 |
| 31845_at | ELF4 | 1.1 | 2.1 | 0.008 | 1.29 | 0.302 | E74-like factor 4 (ets domain transcription factor) |
| 57163_at | ELOVL1 | 1.0 | -1.3 | 0.013 | -1.01 | 0.418 | elongation of very long chain fatty acids (FEN1/Elo2, SUR4/Elo3, yeast)-like 1 |
| 224374_s_at | EMILIN2 | -1.1 | 2.0 | 0.006 | 1.22 | 0.072 | elastin microfibril interfacer 2 /// elastin microfibril interfacer 2 |
| 205521_at | ENDOGL1 | 1.4 | 4.0 | 0.031 | 1.44 | 0.711 | Endonuclease G-like 1 |
| 210839_s_at | ENPP2 | -1.1 | 2.1 | 0.027 | -1.23 | 0.987 | ectonucleotide pyrophosphatase/phosphodiesterase 2 (autotaxin) |
| 212681_at | EPB41L3 | -1.2 | 2.1 | 0.007 | 1.06 | 0.299 | erythrocyte membrane protein band 4.1-like 3 |
| 227609_at | EPSTI1 | 1.7 | 7.6 | 0.005 | 3.32 | 0.177 | epithelial stromal interaction 1 (breast) |
| 205767_at | EREG | 1.1 | -1.2 | 0.042 | 1.05 | 0.056 | epiregulin |
| 204774_at | EVI2A | 1.7 | 3.5 | 0.010 | 2.11 | 0.079 | ecotropic viral integration site 2A |
| 211742_s_at | EVI2B | 1.3 | 3.1 | 0.006 | 1.48 | 0.089 | ecotropic viral integration site 2B /// ecotropic viral integration site 2B |
| 227232_at | EVL | -1.2 | 1.4 | 0.001 | -1.09 | 0.110 | Enah/Vasp-like |
| 225686_at | FAM33A | 1.4 | 2.4 | 0.003 | 1.27 | 0.444 | family with sequence similarity 33, member A |
| 209683_at | FAM49A | 1.6 | 2.5 | 0.006 | 1.55 | 0.429 | Family with sequence similarity 49, member A |
| 217916_s_at | FAM49B | 1.6 | 2.4 | 0.005 | 1.49 | 0.436 | family with sequence similarity 49, member B |
| 218898_at | FAM57A | -1.6 | -2.3 | 0.019 | -1.56 | 0.693 | family with sequence similarity 57, member A |
| 231769_at | FBXO6 | 1.3 | 1.9 | 0.002 | 1.55 | 0.066 | F-box protein 6 |
| 211734_s_at | FCER1A | -1.1 | -2.2 | 0.017 | 1.01 | 0.121 | Fc fragment of IgE, high affinity I, receptor for; alpha polypeptide /// Fc fragment of IgE, high affinity I, receptor for; alpha polypeptide |
| 204232_at | FCER1G | 1.0 | 2.9 | 0.002 | 1.67 | **0.011** | Fc fragment of IgE, high affinity I, receptor for; gamma polypeptide |
| 204007_at | FCGR3B | 1.6 | 2.7 | 0.014 | 1.99 | 0.461 | Fc fragment of IgG, low affinity IIIb, receptor (CD16b) |
| 205237_at | FCN1 | 1.2 | 3.1 | 0.006 | 1.57 | 0.079 | ficolin (collagen/fibrinogen domain containing) 1 |
| 203638_s_at | FGFR2 | -1.8 | -3.8 | 0.024 | -1.82 | 0.112 | fibroblast growth factor receptor 2 (bacteria-expressed kinase, keratinocyte growth factor receptor, craniofacial dysostosis 1, Crouzon syndrome, Pfeiffer syndrome, Jackson-Weiss syndrome) |
| 204834_at | FGL2 | -1.2 | 1.8 | 0.006 | 1.16 | 0.050 | fibrinogen-like 2 |
| 208438_s_at | FGR | 1.8 | 3.0 | 0.011 | 1.97 | 0.937 | Gardner-Rasheed feline sarcoma viral (v-fgr) oncogene homolog |
| 219117_s_at | FKBP11 | 1.5 | 2.9 | 0.000 | 2.04 | **0.009** | FK506 binding protein 11, 19 kDa |
| 215704_at | FLG | -1.1 | -1.5 | 0.011 | -1.13 | 0.242 | filaggrin |
| 204236_at | FLI1 | -1.1 | 2.4 | 0.008 | 1.31 | 0.140 | Friend leukemia virus integration 1 |
| 218614_at | FLJ10652 | 1.1 | 1.9 | 0.037 | 1.21 | 0.992 | hypothetical protein FLJ10652 |
| 218999_at | FLJ11000 | -1.2 | 1.4 | 0.002 | 1.09 | **0.031** | hypothetical protein FLJ11000 |
| 219694_at | FLJ11127 | 1.1 | 2.0 | 0.001 | 1.39 | **0.002** | hypothetical protein FLJ11127 |
| 236565_s_at | FLJ11196 | 1.2 | 2.2 | 0.007 | 1.16 | 0.115 | acheron |
| 218627_at | FLJ11259 | 1.0 | 1.6 | 0.023 | 1.17 | 0.062 | hypothetical protein FLJ11259 |
| 53720_at | FLJ11286 | 1.0 | 2.0 | 0.000 | 1.18 | **0.019** | hypothetical protein FLJ11286 |
| 208918_s_at | FLJ13052 | 1.7 | 3.8 | 0.003 | 2.00 | 0.226 | NAD kinase |
| 218986_s_at | FLJ20035 | -1.2 | 1.9 | 0.000 | -1.13 | 0.640 | hypothetical protein FLJ20035 |
| 219696_at | FLJ20054 | 1.5 | 2.3 | 0.036 | 1.40 | 0.946 | hypothetical protein FLJ20054 |
| 219690_at | FLJ22573 | -1.1 | 1.7 | 0.036 | 1.38 | 0.050 | hypothetical protein FLJ22573 |
| 228152_s_at | FLJ31033 | 1.1 | 4.4 | 0.016 | 1.88 | 0.187 | hypothetical protein FLJ31033 |
| 238429_at | FLJ33069 | -1.1 | 2.2 | 0.042 | 1.30 | 0.596 | hypothetical protein FLJ33069 |
| 204789_at | FMNL1 | -1.0 | 1.5 | 0.020 | 1.33 | 0.438 | formin-like 1 |
| 226184_at | FMNL2 | 1.8 | 2.7 | 0.001 | 2.00 | 0.128 | formin-like 2 |
| 205666_at | FMO1 | -1.4 | -1.8 | 0.041 | -1.37 | 0.280 | flavin containing monooxygenase 1 |
| 229865_at | FNDC3B | -1.1 | 1.6 | 0.006 | -1.03 | **0.029** | fibronectin type III domain containing 3B |
| 205119_s_at | FPR1 | 1.1 | 2.6 | 0.019 | 1.30 | 0.307 | formyl peptide receptor 1 /// formyl peptide receptor 1 |
| 230422_at | FPRL2 | 1.5 | 4.5 | 0.011 | 1.87 | 0.230 | formyl peptide receptor-like 2 |
| 203698_s_at | FRZB | 1.5 | -2.0 | 0.016 | 1.02 | 0.195 | frizzled-related protein |
| 207345_at | FST | 1.1 | 1.3 | 0.045 | 1.19 | 0.428 | follistatin |
| 211795_s_at | FYB | 1.6 | 6.5 | 0.002 | 1.78 | 0.480 | FYN binding protein (FYB-120/130) |
| 210220_at | FZD2 | 1.2 | 3.7 | 0.037 | 2.22 | **0.007** | frizzled homolog 2 (Drosophila) |
| 205483_s_at | G1P2 | 1.4 | 28.3 | 0.000 | 2.10 | **0.032** | interferon, alpha-inducible protein (clone IFI-15K) |
| 204415_at | G1P3 | 1.4 | 7.1 | 0.001 | 2.17 | 0.242 | interferon, alpha-inducible protein (clone IFI-6-16) |
| 218313_s_at | GALNT7 | 1.5 | 2.8 | 0.006 | 1.37 | 0.862 | UDP-N-acetyl-alpha-D-galactosamine:polypeptide N-acetylgalactosaminyltransferase 7 (GalNAc-T7) |
| 204457_s_at | GAS1 | -1.5 | -2.7 | 0.014 | -1.36 | 0.381 | growth arrest-specific 1 |
| 202270_at | GBP1 | 1.5 | 8.2 | 0.000 | 2.33 | 0.083 | guanylate binding protein 1, interferon-inducible, 67kDa /// guanylate binding protein 1, interferon-inducible, 67kDa |
| 242907_at | GBP2 | 1.0 | 1.7 | 0.012 | 1.21 | 0.294 | guanylate binding protein 2, interferon-inducible |
| 235574_at | GBP4 | 1.5 | 4.1 | 0.014 | 2.63 | 0.577 | guanylate binding protein 4 |
| 203765_at | GCA | 1.2 | 2.6 | 0.002 | 1.36 | 0.193 | grancalcin, EF-hand calcium binding protein /// grancalcin, EF-hand calcium binding protein |
| 204224_s_at | GCH1 | 1.5 | 2.8 | 0.011 | 1.28 | 0.598 | GTP cyclohydrolase 1 (dopa-responsive dystonia) |
| 219243_at | GIMAP4 | -1.1 | 3.7 | 0.000 | 1.19 | 0.079 | GTPase, IMAP family member 4 |
| 218805_at | GIMAP5 | 1.0 | 2.8 | 0.001 | 1.13 | 0.075 | GTPase, IMAP family member 5 /// GTPase, IMAP family member 5 |
| 219777_at | GIMAP6 | -1.3 | 2.9 | 0.002 | 1.15 | 0.061 | GTPase, IMAP family member 6 |
| 228071_at | GIMAP7 | 1.3 | 2.7 | 0.044 | 1.46 | 0.613 | GTPase, IMAP family member 7 |
| 235306_at | GIMAP8 | -1.2 | 2.3 | 0.000 | -1.03 | 0.053 | GTPase, IMAP family member 8 |
| 214085_x_at | GLIPR1 | 1.0 | 1.7 | 0.035 | 1.30 | 0.216 | GLI pathogenesis-related 1 (glioma) |
| 229435_at | GLIS3 | 1.0 | 1.9 | 0.003 | 1.10 | 0.185 | GLIS family zinc finger 3 |
| 217807_s_at | GLTSCR2 | -1.9 | -2.3 | 0.016 | -1.73 | 0.227 | glioma tumor suppressor candidate region gene 2 |
| 242281_at | GLUL | 1.4 | 2.6 | 0.017 | 1.78 | 0.069 | Glutamate-ammonia ligase (glutamine synthase) |
| 204220_at | GMFG | 1.3 | 3.9 | 0.034 | 1.88 | **0.046** | glia maturation factor, gamma |
| 37145_at | GNLY | 1.3 | 13.3 | 0.011 | 1.30 | 0.796 | granulysin |
| 223423_at | GPR160 | 1.3 | 2.1 | 0.029 | 1.53 | 0.258 | G protein-coupled receptor 160 |
| 207651_at | GPR171 | 1.9 | 5.4 | 0.000 | 1.88 | **0.002** | G protein-coupled receptor 171 |
| 210279_at | GPR18 | 2.0 | 4.8 | 0.004 | 2.81 | 0.089 | G protein-coupled receptor 18 |
| 214467_at | GPR65 | 1.9 | 5.5 | 0.001 | 1.95 | 0.252 | G protein-coupled receptor 65 |
| 215075_s_at | GRB2 | 1.2 | 1.9 | 0.045 | 1.26 | 0.269 | growth factor receptor-bound protein 2 |
| 205488_at | GZMA | 1.3 | 5.4 | 0.002 | 2.16 | 0.059 | granzyme A (granzyme 1, cytotoxic T-lymphocyte-associated serine esterase 3) /// granzyme A (granzyme 1, cytotoxic T-lymphocyte-associated serine esterase 3) |
| 206666_at | GZMK | 1.3 | 7.0 | 0.015 | 2.33 | 0.277 | granzyme K (serine protease, granzyme 3; tryptase II) /// granzyme K (serine protease, granzyme 3; tryptase II) |
| 212873_at | HA-1 | 1.3 | 2.5 | 0.011 | 1.46 | 0.098 | minor histocompatibility antigen HA-1 |
| 235458_at | HAVCR2 | 1.1 | 13.5 | 0.001 | 1.75 | 0.123 | hepatitis A virus cellular receptor 2 |
| 218663_at | HCAP-G | 1.6 | 2.8 | 0.025 | 1.75 | 0.999 | chromosome condensation protein G |
| 208018_s_at | HCK | 1.4 | 2.8 | 0.013 | 1.38 | 0.484 | hemopoietic cell kinase |
| 202957_at | HCLS1 | 1.3 | 3.0 | 0.009 | 1.85 | 0.072 | hematopoietic cell-specific Lyn substrate 1 |
| 223640_at | HCST | 1.1 | 3.6 | 0.000 | 1.66 | **0.005** | hematopoietic cell signal transducer |
| 219863_at | HERC5 | 1.0 | 3.4 | 0.000 | 1.61 | **0.005** | hect domain and RLD 5 |
| 219352_at | HERC6 | 1.4 | 7.3 | 0.000 | 2.58 | **0.029** | hect domain and RLD 6 |
| 213374_x_at | HIBCH | 1.4 | -1.1 | 0.020 | 1.31 | 0.960 | 3-hydroxyisobutyryl-Coenzyme A hydrolase |
| 209806_at | HIST1H2BK | 1.4 | 2.1 | 0.009 | 1.48 | 0.420 | histone 1, H2bk |
| 214290_s_at | HIST2H2AA | 1.5 | 1.9 | 0.028 | 1.09 | **0.046** | histone 2, H2aa |
| 213537_at | HLA-DPA1 | 1.5 | 2.1 | 0.041 | 1.65 | 0.086 | major histocompatibility complex, class II, DP alpha 1 |
| 200904_at | HLA-E | -1.8 | -1.1 | 0.004 | -1.28 | **0.036** | major histocompatibility complex, class I, E |
| 207165_at | HMMR | 1.5 | 2.4 | 0.005 | 1.62 | 0.991 | hyaluronan-mediated motility receptor (RHAMM) |
| 217755_at | HN1 | 2.0 | 2.8 | 0.023 | 2.00 | 0.837 | hematological and neurological expressed 1 |
| 211732_x_at | HNMT | -1.1 | 1.3 | 0.004 | 1.03 | 0.067 | histamine N-methyltransferase /// histamine N-methyltransferase |
| 211597_s_at | HOP | -1.5 | -3.3 | 0.005 | -1.51 | 0.058 | homeodomain-only protein /// homeodomain-only protein |
| 202854_at | HPRT1 | 1.2 | 1.6 | 0.008 | 1.25 | 0.461 | hypoxanthine phosphoribosyltransferase 1 (Lesch-Nyhan syndrome) |
| 205466_s_at | HS3ST1 | 1.9 | 2.8 | 0.037 | 1.77 | 0.073 | heparan sulfate (glucosamine) 3-O-sulfotransferase 1 |
| 211538_s_at | HSPA2 | -1.7 | -2.9 | 0.038 | -1.65 | 0.351 | heat shock 70kDa protein 2 |
| 206133_at | HSXIAPAF1 | 1.3 | 6.3 | 0.000 | 1.73 | 0.102 | XIAP associated factor-1 |
| 226711_at | HTLF | 1.2 | 2.2 | 0.002 | 1.22 | 0.871 | human T-cell leukemia virus enhancer factor |
| 202638_s_at | ICAM1 | 1.1 | 2.0 | 0.005 | 1.13 | 0.470 | intercellular adhesion molecule 1 (CD54), human rhinovirus receptor |
| 213620_s_at | ICAM2 | -1.0 | 2.7 | 0.003 | 1.36 | **0.045** | intercellular adhesion molecule 2 |
| 203327_at | IDE | -1.2 | -2.4 | 0.029 | -1.22 | 0.379 | insulin-degrading enzyme |
| 206332_s_at | IFI16 | 1.7 | 2.6 | 0.002 | 1.71 | 0.326 | interferon, gamma-inducible protein 16 |
| 209417_s_at | IFI35 | -1.0 | 3.8 | 0.000 | 1.45 | **0.029** | interferon-induced protein 35 |
| 214453_s_at | IFI44 | 1.7 | 12.6 | 0.000 | 3.12 | **0.009** | interferon-induced protein 44 |
| 204439_at | IFI44L | 1.0 | 16.3 | 0.096 | 2.81 | **0.006** | interferon-induced protein 44-like |
| 219209_at | IFIH1 | 1.2 | 5.7 | 0.022 | 1.95 | 0.132 | interferon induced with helicase C domain 1 |
| 203153_at | IFIT1 | 1.4 | 19.8 | 0.014 | 2.68 | 0.317 | interferon-induced protein with tetratricopeptide repeats 1 /// interferon-induced protein with tetratricopeptide repeats 1 |
| 226757_at | IFIT2 | -1.2 | 7.4 | 0.000 | 1.21 | 0.067 | interferon-induced protein with tetratricopeptide repeats 2 |
| 204747_at | IFIT3 | 1.0 | 4.9 | 0.001 | 1.31 | **0.047** | interferon-induced protein with tetratricopeptide repeats 3 |
| 203595_s_at | IFIT5 | 1.1 | 2.4 | 0.002 | 1.22 | 0.252 | interferon-induced protein with tetratricopeptide repeats 5 |
| 214022_s_at | IFITM1 | 1.2 | 3.8 | 0.000 | 1.59 | **0.014** | interferon induced transmembrane protein 1 (9-27) |
| 208448_x_at | IFNA16 | 1.0 | 1.4 | 0.036 | 1.11 | 0.112 | interferon, alpha 16 |
| 217148_x_at | IGLV2-14 | 1.2 | 2.6 | 0.029 | 2.50 | **0.018** | immunoglobulin lambda variable 2-14 |
| 206420_at | IGSF6 | 1.6 | 6.7 | 0.001 | 2.35 | **0.036** | immunoglobulin superfamily, member 6 |
| 204912_at | IL10RA | 1.3 | 3.4 | 0.000 | 1.49 | **0.030** | interleukin 10 receptor, alpha |
| 222868_s_at | IL18BP | 1.2 | 2.1 | 0.006 | 1.24 | 0.596 | interleukin 18 binding protein |
| 207072_at | IL18RAP | 1.1 | 3.0 | 0.001 | 1.39 | 0.233 | interleukin 18 receptor accessory protein |
| 207526_s_at | IL1RL1 | 1.3 | 3.0 | 0.002 | 1.79 | 0.075 | interleukin 1 receptor-like 1 |
| 219115_s_at | IL20RA | -1.3 | -1.4 | 0.048 | -1.29 | 0.519 | interleukin 20 receptor, alpha |
| 221658_s_at | IL21R | 1.2 | 8.6 | 0.000 | 2.36 | **0.043** | interleukin 21 receptor |
| 210313_at | ILT7 | 1.2 | 5.0 | 0.001 | 1.35 | 0.566 | leukocyte immunoglobulin-like receptor, subfamily A (without TM domain), member 4 |
| 203819_s_at | IMP-3 | -1.6 | 2.6 | 0.001 | -1.46 | 0.052 | IGF-II mRNA-binding protein 3 |
| 210029_at | INDO | 1.4 | 4.1 | 0.001 | 1.67 | 0.925 | indoleamine-pyrrole 2,3 dioxygenase |
| 227087_at | INPP4A | 1.1 | 1.6 | 0.031 | 1.23 | **0.027** | Inositol polyphosphate-4-phosphatase, type I, 107kDa |
| 231779_at | IRAK2 | 1.3 | 1.9 | 0.000 | 1.23 | 0.747 | interleukin-1 receptor-associated kinase 2 |
| 202531_at | IRF1 | 1.2 | 2.1 | 0.001 | 1.40 | 0.347 | interferon regulatory factor 1 |
| 208436_s_at | IRF7 | 1.8 | 7.6 | 0.000 | 2.22 | **0.027** | interferon regulatory factor 7 |
| 204057_at | IRF8 | 1.9 | 5.7 | 0.028 | 1.93 | 0.344 | interferon regulatory factor 8 /// interferon regulatory factor 8 |
| 33304_at | ISG20 | 1.3 | 6.1 | 0.000 | 1.73 | 0.385 | interferon stimulated gene 20kDa |
| 213416_at | ITGA4 | 1.5 | 7.4 | 0.022 | 2.60 | 0.845 | integrin, alpha 4 (antigen CD49D, alpha 4 subunit of VLA-4 receptor) |
| 213475_s_at | ITGAL | 1.2 | 3.8 | 0.041 | 1.56 | 0.097 | integrin, alpha L (antigen CD11A (p180), lymphocyte function-associated antigen 1; alpha polypeptide) |
| 202803_s_at | ITGB2 | 1.2 | 3.1 | 0.012 | 1.67 | 0.174 | integrin, beta 2 (antigen CD18 (p95), lymphocyte function-associated antigen 1; macrophage antigen 1 (mac-1) beta subunit) |
| 205422_s_at | ITGBL1 | -1.0 | -3.3 | 0.018 | 1.21 | 0.306 | integrin, beta-like 1 (with EGF-like repeat domains) |
| 211339_s_at | ITK | 1.4 | 1.9 | 0.022 | 1.21 | 0.839 | IL2-inducible T-cell kinase |
| 202746_at | ITM2A | -1.3 | -1.9 | 0.028 | -1.13 | 0.906 | integral membrane protein 2A |
| 202503_s_at | KIAA0101 | 1.8 | 2.6 | 0.001 | 1.92 | 0.625 | KIAA0101 |
| 204157_s_at | KIAA0999 | -1.1 | 1.3 | 0.024 | 1.41 | **0.008** | KIAA0999 protein |
| 212327_at | KIAA1102 | -1.5 | -2.5 | 0.016 | -1.59 | 0.621 | KIAA1102 protein |
| 212942_s_at | KIAA1199 | 1.8 | 1.4 | 0.041 | 1.69 | 0.489 | KIAA1199 |
| 225076_s_at | KIAA1404 | 1.0 | 2.0 | 0.003 | 1.47 | 0.122 | KIAA1404 protein |
| 241347_at | KIAA1618 | -1.0 | 2.0 | 0.001 | 1.13 | 0.109 | KIAA1618 |
| 206364_at | KIF14 | 1.2 | 1.9 | 0.035 | 1.55 | 0.100 | kinesin family member 14 |
| 209212_s_at | KLF5 | -1.6 | -2.4 | 0.043 | -1.59 | 0.693 | Kruppel-like factor 5 (intestinal) |
| 206785_s_at | KLRC1 /// KLRC2 | 1.1 | 31.6 | 0.000 | 1.94 | 0.077 | killer cell lectin-like receptor subfamily C, member 1 /// killer cell lectin-like receptor subfamily C, member 2 |
| 220646_s_at | KLRF1 | 1.2 | 5.3 | 0.001 | 1.37 | 0.319 | killer cell lectin-like receptor subfamily F, member 1 |
| 205821_at | KLRK1 | 1.1 | 2.4 | 0.018 | 1.48 | 0.056 | killer cell lectin-like receptor subfamily K, member 1 |
| 205306_x_at | KMO | 1.4 | 2.0 | 0.011 | 1.51 | 0.434 | kynurenine 3-monooxygenase (kynurenine 3-hydroxylase) |
| 218963_s_at | KRT23 | -1.4 | -3.0 | 0.014 | -1.80 | **0.045** | keratin 23 (histone deacetylase inducible) |
| 207908_at | KRT2A | -1.3 | -4.1 | 0.018 | -1.25 | 0.821 | keratin 2A (epidermal ichthyosis bullosa of Siemens) |
| 206486_at | LAG3 | -1.1 | 4.9 | 0.000 | 1.44 | **0.036** | lymphocyte-activation gene 3 |
| 210644_s_at | LAIR1 | 1.1 | 2.5 | 0.001 | 1.30 | **0.014** | leukocyte-associated Ig-like receptor 1 |
| 205569_at | LAMP3 | 1.6 | 2.5 | 0.008 | 1.80 | 0.524 | lysosomal-associated membrane protein 3 |
| 217933_s_at | LAP3 | 1.4 | 2.4 | 0.001 | 1.47 | 0.834 | leucine aminopeptidase 3 |
| 201721_s_at | LAPTM5 | 1.5 | 3.9 | 0.001 | 2.06 | **0.029** | lysosomal associated multispanning membrane protein 5 |
| 213261_at | LBA1 | 1.2 | 1.7 | 0.000 | 1.28 | 0.393 | lupus brain antigen 1 |
| 207710_at | LCE2B | -1.8 | -2.5 | 0.038 | -1.84 | 0.462 | late cornified envelope 2B |
| 204890_s_at | LCK | 1.3 | 4.6 | 0.009 | 1.27 | 0.409 | lymphocyte-specific protein tyrosine kinase |
| 205270_s_at | LCP2 | 1.3 | 4.9 | 0.047 | 1.78 | 0.698 | lymphocyte cytosolic protein 2 (SH2 domain containing leukocyte protein of 76kDa) |
| 208450_at | LGALS2 | 1.5 | 4.4 | 0.004 | 2.22 | **0.025** | lectin, galactoside-binding, soluble, 2 (galectin 2) /// lectin, galactoside-binding, soluble, 2 (galectin 2) |
| 200923_at | LGALS3BP | 1.4 | 3.4 | 0.001 | 2.03 | 0.177 | lectin, galactoside-binding, soluble, 3 binding protein |
| 203236_s_at | LGALS9 | 1.6 | 5.6 | 0.011 | 2.25 | 0.090 | lectin, galactoside-binding, soluble, 9 (galectin 9) |
| 201212_at | LGMN | 1.1 | 2.9 | 0.001 | 1.37 | **0.020** | legumain |
| 219364_at | LGP2 | -1.1 | 2.2 | 0.002 | 1.06 | 0.157 | likely ortholog of mouse D11lgp2 |
| 210660_at | LILRA1 | 1.0 | 4.5 | 0.003 | 1.16 | 0.378 | leukocyte immunoglobulin-like receptor, subfamily A (with TM domain), member 1 /// leukocyte immunoglobulin-like receptor, subfamily A (with TM domain), member 1 |
| 211133_x_at | LILRB2 /// LILRB3 | -1.1 | 3.2 | 0.000 | 1.04 | 0.574 | leukocyte immunoglobulin-like receptor, subfamily B (with TM and ITIM domains), member 2 /// leukocyte immunoglobulin-like receptor, subfamily B (with TM and ITIM domains), member 3 |
| 203320_at | LNK | 1.2 | 2.8 | 0.000 | 1.58 | **0.006** | lymphocyte adaptor protein |
| 230061_at | LOC116441 | 1.6 | 3.4 | 0.012 | 2.16 | 0.551 | Hypothetical protein BC014339 |
| 226329_s_at | LOC129531 | 1.3 | 1.6 | 0.048 | 1.29 | 0.059 | hypothetical protein BC018453 |
| 226702_at | LOC129607 | 1.3 | 11.8 | 0.002 | 2.27 | 0.302 | hypothetical protein LOC129607 |
| 228654_at | LOC139886 | 1.7 | 2.5 | 0.041 | 1.42 | 0.237 | hypothetical protein LOC139886 |
| 238476_at | LOC153222 | -1.5 | 1.1 | 0.007 | -1.14 | 0.392 | adult retina protein |
| 236769_at | LOC158402 | 1.4 | 1.9 | 0.026 | 1.17 | 0.100 | hypothetical protein LOC158402 |
| 225541_at | LOC200916 | 1.1 | 1.7 | 0.017 | 1.29 | 0.082 | similar to ribosomal protein L22 |
| 226219_at | LOC257106 | 1.2 | 1.7 | 0.022 | 1.06 | 0.379 | hypothetical protein LOC257106 |
| 225767_at | LOC284801 | 1.0 | 1.9 | 0.002 | 1.22 | 0.278 | hypothetical protein LOC284801 |
| 225033_at | LOC286167 | 1.0 | 1.8 | 0.012 | 1.02 | 0.364 | hypothetical protein LOC286167 |
| 216565_x_at | LOC391020 | 1.0 | 2.6 | 0.001 | 1.27 | 0.153 | similar to Interferon-induced transmembrane protein 3 (Interferon-inducible protein 1-8U) |
| 230405_at | LOC441109 | 1.1 | 5.5 | 0.000 | 1.58 | 0.093 | hypothetical gene supported by AL713721 |
| 227628_at | LOC493869 | 1.0 | 1.8 | 0.002 | 1.35 | 0.128 | similar to RIKEN cDNA 2310016C16 |
| 213142_x_at | LOC54103 | 1.0 | 1.9 | 0.002 | -1.15 | 0.990 | hypothetical protein LOC54103 |
| 214791_at | LOC93349 | 1.1 | 2.7 | 0.001 | 1.48 | **0.034** | hypothetical protein BC004921 |
| 207720_at | LOR | -1.3 | -1.5 | 0.019 | -1.17 | 0.923 | loricrin |
| 244300_at | LPHN3 | 1.8 | -1.1 | 0.000 | 2.02 | 0.811 | Latrophilin 3 |
| 216250_s_at | LPXN | 1.3 | 3.5 | 0.002 | 1.60 | 0.294 | leupaxin |
| 220532_s_at | LR8 | 1.2 | 1.9 | 0.002 | 1.48 | 0.302 | LR8 protein |
| 227462_at | LRAP | 1.0 | 1.5 | 0.004 | 1.22 | 0.263 | Leukocyte-derived arginine aminopeptidase |
| 202145_at | LY6E | 1.1 | 2.7 | 0.002 | 1.39 | **0.011** | lymphocyte antigen 6 complex, locus E |
| 206584_at | LY96 | 1.2 | 4.9 | 0.000 | 2.08 | **0.039** | lymphocyte antigen 96 |
| 202626_s_at | LYN | 1.6 | 4.0 | 0.008 | 1.77 | 0.244 | v-yes-1 Yamaguchi sarcoma viral related oncogene homolog /// v-yes-1 Yamaguchi sarcoma viral related oncogene homolog |
| 218437_s_at | LZTFL1 | -1.4 | -2.0 | 0.016 | -1.69 | **0.009** | leucine zipper transcription factor-like 1 |
| 219574_at | MARCH1 | 1.2 | 2.6 | 0.002 | 1.53 | **0.008** | membrane-associated ring finger (C3HC4) 1 |
| 228468_at | MASTL | 1.2 | 1.8 | 0.008 | 1.15 | 0.252 | microtubule associated serine/threonine kinase-like |
| 202350_s_at | MATN2 | -1.8 | -4.9 | 0.001 | -1.83 | 0.430 | matrilin 2 |
| 204825_at | MELK | 1.7 | 2.7 | 0.000 | 1.39 | 0.727 | maternal embryonic leucine zipper kinase |
| 210493_s_at | MFAP3L | 1.4 | 1.0 | 0.021 | 1.44 | 0.189 | microfibrillar-associated protein 3-like |
| 236285_at | MGC16635 | 1.7 | 4.3 | 0.005 | 1.36 | 0.796 | Hypothetical protein BC009980 |
| 228439_at | MGC20410 | 1.0 | 2.8 | 0.012 | 1.08 | 0.805 | hypothetical protein BC012330 |
| 226748_at | MGC35274 | 1.2 | 2.2 | 0.000 | 1.34 | 0.532 | hypothetical protein MGC35274 |
| 225799_at | MGC4677 /// LOC541471 | 1.3 | 3.1 | 0.005 | 1.58 | 0.576 | hypothetical protein MGC4677 /// hypothetical LOC541471 protein |
| 226039_at | MGC52110 | 1.3 | 3.8 | 0.000 | 1.26 | 0.111 | Hypothetical protein MGC52110 |
| 227983_at | MGC7036 | -1.1 | 1.7 | 0.001 | -1.01 | 0.067 | hypothetical protein MGC7036 |
| 206247_at | MICB | 1.3 | 4.2 | 0.000 | 1.89 | **0.021** | MHC class I polypeptide-related sequence B |
| 238025_at | MLKL | 1.6 | 2.3 | 0.039 | 1.99 | 0.715 | mixed lineage kinase domain-like |
| 219607_s_at | MS4A4A | 1.2 | 5.1 | 0.001 | 1.62 | 0.060 | membrane-spanning 4-domains, subfamily A, member 4 |
| 220346_at | MTHFD2L | 1.2 | -1.3 | 0.014 | -1.09 | 0.769 | methylenetetrahydrofolate dehydrogenase (NADP+ dependent) 2-like |
| 202086_at | MX1 | 1.2 | 11.5 | 0.000 | 2.28 | **0.041** | myxovirus (influenza virus) resistance 1, interferon-inducible protein p78 (mouse) /// myxovirus (influenza virus) resistance 1, interferon-inducible protein p78 (mouse) |
| 204994_at | MX2 | 1.0 | 4.8 | 0.000 | 1.42 | 0.071 | myxovirus (influenza virus) resistance 2 (mouse) |
| 213906_at | MYBL1 | 1.3 | 1.9 | 0.015 | 1.20 | 0.648 | v-myb myeloblastosis viral oncogene homolog (avian)-like 1 |
| 219368_at | NAP1L2 | -1.3 | -1.7 | 0.050 | -1.62 | **0.022** | nucleosome assembly protein 1-like 2 |
| 228056_s_at | NAPSB | 1.8 | 10.8 | 0.016 | 5.21 | 0.067 | napsin B aspartic peptidase pseudogene |
| 202906_s_at | NBS1 | 1.3 | 1.8 | 0.021 | 1.31 | 0.595 | Nijmegen breakage syndrome 1 (nibrin) |
| 209949_at | NCF2 | -1.0 | 1.9 | 0.000 | 1.05 | 0.209 | neutrophil cytosolic factor 2 (65kDa, chronic granulomatous disease, autosomal 2) |
| 207677_s_at | NCF4 | -1.1 | 3.1 | 0.010 | 1.71 | 0.117 | neutrophil cytosolic factor 4, 40kDa /// neutrophil cytosolic factor 4, 40kDa |
| 213915_at | NKG7 | 1.2 | 9.6 | 0.001 | 2.04 | 0.162 | natural killer cell group 7 sequence |
| 203964_at | NMI | 1.9 | 3.0 | 0.001 | 1.58 | 0.567 | N-myc (and STAT) interactor |
| 226474_at | NOD27 | -1.1 | 1.8 | 0.034 | 1.33 | **0.048** | nucleotide-binding oligomerization domains 27 |
| 209959_at | NR4A3 | 1.6 | 2.3 | 0.043 | 1.27 | 0.196 | nuclear receptor subfamily 4, group A, member 3 |
| 223298_s_at | NT5C3 | 1.8 | 2.5 | 0.018 | 1.39 | 0.141 | 5'-nucleotidase, cytosolic III |
| 203939_at | NT5E | -1.3 | 2.1 | 0.001 | -1.46 | 0.367 | 5'-nucleotidase, ecto (CD73) |
| 202869_at | OAS1 | 1.9 | 6.9 | 0.000 | 2.83 | **0.021** | 2',5'-oligoadenylate synthetase 1, 40/46kDa |
| 204972_at | OAS2 | 1.2 | 3.7 | 0.000 | 1.52 | **0.029** | 2'-5'-oligoadenylate synthetase 2, 69/71kDa |
| 218400_at | OAS3 | 1.4 | 3.9 | 0.000 | 1.47 | 0.422 | 2'-5'-oligoadenylate synthetase 3, 100kDa |
| 205660_at | OASL | 1.6 | 5.1 | 0.000 | 1.96 | 0.062 | 2'-5'-oligoadenylate synthetase-like |
| 212768_s_at | OLFM4 | 1.2 | 4.0 | 0.033 | 2.16 | 0.184 | olfactomedin 4 |
| 213125_at | OLFML2B | 1.0 | 2.2 | 0.009 | 1.27 | **0.020** | olfactomedin-like 2B |
| 214615_at | P2RY10 | 1.1 | 2.0 | 0.032 | 1.25 | 0.111 | purinergic receptor P2Y, G-protein coupled, 10 |
| 229138_at | PARP11 | 1.2 | 1.8 | 0.016 | 1.27 | 0.072 | poly (ADP-ribose) polymerase family, member 11 |
| 224701_at | PARP14 | 1.5 | 3.7 | 0.000 | 1.48 | 0.262 | poly (ADP-ribose) polymerase family, member 14 |
| 223220_s_at | PARP9 | 1.3 | 3.5 | 0.003 | 1.87 | 0.763 | poly (ADP-ribose) polymerase family, member 9 |
| 219148_at | PBK | 1.6 | 2.5 | 0.011 | 1.60 | 0.389 | PDZ binding kinase |
| 203803_at | PCYOX1 | -1.2 | -2.1 | 0.004 | -1.46 | 0.052 | prenylcysteine oxidase 1 |
| 227458_at | PDCD1LG1 | 1.4 | 2.7 | 0.010 | 1.48 | 0.535 | CD274 antigen |
| 222317_at | PDE3B | 1.1 | 3.1 | 0.004 | 1.32 | 0.187 | Phosphodiesterase 3B, cGMP-inhibited |
| 203708_at | PDE4B | 1.3 | 4.1 | 0.030 | 1.15 | 0.708 | phosphodiesterase 4B, cAMP-specific (phosphodiesterase E4 dunce homolog, Drosophila) |
| 223619_x_at | PECR | 1.5 | -1.0 | 0.015 | 1.39 | 0.733 | peroxisomal trans-2-enoyl-CoA reductase |
| 227068_at | PGK1 | 1.6 | 2.4 | 0.015 | 1.57 | 0.323 | phosphoglycerate kinase 1 |
| 213227_at | PGRMC2 | -1.5 | -2.4 | 0.016 | -1.51 | 0.788 | progesterone receptor membrane component 2 |
| 217997_at | PHLDA1 | 1.6 | 2.2 | 0.032 | 1.09 | 0.255 | pleckstrin homology-like domain, family A, member 1 |
| 226459_at | PIK3AP1 | 1.4 | 5.3 | 0.025 | 1.72 | 0.273 | phosphoinositide-3-kinase adaptor protein 1 |
| 203879_at | PIK3CD | 1.6 | 3.6 | 0.001 | 1.88 | 0.134 | phosphoinositide-3-kinase, catalytic, delta polypeptide /// phosphoinositide-3-kinase, catalytic, delta polypeptide |
| 212249_at | PIK3R1 | -1.3 | 1.1 | 0.001 | 1.05 | **0.003** | phosphoinositide-3-kinase, regulatory subunit 1 (p85 alpha) |
| 222218_s_at | PILRA | -1.2 | 3.0 | 0.036 | 1.20 | 0.190 | paired immunoglobin-like type 2 receptor alpha |
| 221605_s_at | PIPOX | 1.2 | 1.6 | 0.001 | 1.34 | **0.038** | pipecolic acid oxidase |
| 221854_at | PKP1 | -1.3 | -2.6 | 0.025 | -1.56 | 0.535 | plakophilin 1 (ectodermal dysplasia/skin fragility syndrome) |
| 219014_at | PLAC8 | 1.7 | 11.0 | 0.001 | 2.38 | 0.528 | placenta-specific 8 |
| 210845_s_at | PLAUR | 1.2 | 2.4 | 0.003 | 1.56 | 0.237 | plasminogen activator, urokinase receptor |
| 213309_at | PLCL2 | -1.3 | 1.9 | 0.045 | 1.39 | 0.081 | phospholipase C-like 2 |
| 203470_s_at | PLEK | 1.8 | 8.4 | 0.001 | 2.69 | **0.012** | pleckstrin |
| 202620_s_at | PLOD2 | 1.3 | 2.5 | 0.003 | 1.34 | 0.430 | procollagen-lysine, 2-oxoglutarate 5-dioxygenase 2 |
| 210198_s_at | PLP1 | -1.7 | -3.1 | 0.044 | -1.37 | 0.798 | proteolipid protein 1 (Pelizaeus-Merzbacher disease, spastic paraplegia 2, uncomplicated) |
| 202446_s_at | PLSCR1 | 1.5 | 5.3 | 0.001 | 1.83 | 0.814 | phospholipid scramblase 1 |
| 211012_s_at | PML | -1.1 | 4.5 | 0.002 | 1.24 | **0.023** | promyelocytic leukemia |
| 225291_at | PNPT1 | 1.8 | 2.7 | 0.014 | 1.58 | 0.084 | polyribonucleotide nucleotidyltransferase 1 |
| 219756_s_at | POF1B | -1.4 | -2.3 | 0.025 | -1.73 | 0.090 | premature ovarian failure, 1B |
| 210809_s_at | POSTN | -1.3 | -2.4 | 0.020 | -1.01 | 0.626 | periostin, osteoblast specific factor |
| 244011_at | PPM1K | 1.2 | 2.4 | 0.047 | 1.39 | 0.299 | protein phosphatase 1K (PP2C domain containing) |
| 214617_at | PRF1 | 1.0 | 2.3 | 0.002 | 1.23 | 0.204 | perforin 1 (pore forming protein) /// perforin 1 (pore forming protein) |
| 201858_s_at | PRG1 | 1.8 | 8.2 | 0.008 | 2.04 | 0.867 | proteoglycan 1, secretory granule |
| 207808_s_at | PROS1 | -1.4 | -2.3 | 0.003 | -1.39 | 0.375 | protein S (alpha) |
| 219183_s_at | PSCD4 | 1.0 | 2.5 | 0.002 | 1.39 | **0.035** | pleckstrin homology, Sec7 and coiled-coil domains 4 |
| 209606_at | PSCDBP | 1.3 | 3.7 | 0.010 | 1.43 | 0.850 | pleckstrin homology, Sec7 and coiled-coil domains, binding protein /// pleckstrin homology, Sec7 and coiled-coil domains, binding protein |
| 202659_at | PSMB10 | 1.3 | 2.5 | 0.015 | 1.43 | 0.060 | proteasome (prosome, macropain) subunit, beta type, 10 |
| 204279_at | PSMB9 | 1.1 | 2.5 | 0.003 | 1.35 | 0.061 | proteasome (prosome, macropain) subunit, beta type, 9 (large multifunctional protease 2) |
| 201762_s_at | PSME2 | 1.4 | 2.0 | 0.007 | 1.58 | 0.078 | proteasome (prosome, macropain) activator subunit 2 (PA28 beta) |
| 213933_at | PTGER3 | 1.0 | -2.3 | 0.013 | 1.02 | 0.287 | Prostaglandin E receptor 3 (subtype EP3) |
| 209466_x_at | PTN | 1.7 | 2.1 | 0.012 | 1.95 | **0.025** | pleiotrophin (heparin binding growth factor 8, neurite growth-promoting factor 1) |
| 204201_s_at | PTPN13 | -1.8 | -3.1 | 0.001 | -2.22 | **0.010** | protein tyrosine phosphatase, non-receptor type 13 (APO-1/CD95 (Fas)-associated phosphatase) |
| 236539_at | PTPN22 | 2.0 | 3.4 | 0.033 | 2.48 | 0.468 | protein tyrosine phosphatase, non-receptor type 22 (lymphoid) |
| 212588_at | PTPRC | 1.6 | 3.6 | 0.021 | 2.14 | 0.328 | protein tyrosine phosphatase, receptor type, C |
| 219622_at | RAB20 | 1.3 | 2.3 | 0.009 | 1.48 | **0.024** | RAB20, member RAS oncogene family |
| 225064_at | RABEP1 | 1.2 | 1.9 | 0.007 | 1.30 | 0.238 | rabaptin, RAB GTPase binding effector protein 1 |
| 213982_s_at | RABGAP1L | -1.3 | 1.4 | 0.015 | 1.26 | 0.060 | RAB GTPase activating protein 1-like |
| 213603_s_at | RAC2 | 1.5 | 3.6 | 0.013 | 1.93 | 0.110 | ras-related C3 botulinum toxin substrate 2 (rho family, small GTP binding protein Rac2) |
| 204146_at | RAD51AP1 | 1.6 | 2.4 | 0.001 | 1.59 | 0.568 | RAD51 associated protein 1 |
| 206391_at | RARRES1 | 1.5 | 4.7 | 0.018 | 3.14 | 0.091 | retinoic acid receptor responder (tazarotene induced) 1 |
| 204070_at | RARRES3 | -1.2 | 2.9 | 0.001 | 1.23 | 0.058 | retinoic acid receptor responder (tazarotene induced) 3 |
| 205407_at | RECK | -2.0 | -3.2 | 0.027 | -1.99 | **0.012** | reversion-inducing-cysteine-rich protein with kazal motifs |
| 204127_at | RFC3 | 1.1 | 1.6 | 0.003 | 1.19 | 0.764 | replication factor C (activator 1) 3, 38kDa |
| 209568_s_at | RGL1 | -1.1 | 1.9 | 0.002 | 1.13 | **0.025** | ral guanine nucleotide dissociation stimulator-like 1 |
| 213566_at | RNASE6 | 1.2 | 4.2 | 0.011 | 1.80 | **0.015** | ribonuclease, RNase A family, k6 /// ribonuclease, RNase A family, k6 |
| 218738_s_at | RNF138 | 1.2 | 2.0 | 0.036 | 1.21 | 0.091 | ring finger protein 138 |
| 210479_s_at | RORA | -1.6 | -2.3 | 0.006 | -1.46 | 0.312 | RAR-related orphan receptor A |
| 201206_s_at | RRBP1 | -1.0 | 1.4 | 0.012 | 1.05 | 0.141 | ribosome binding protein 1 homolog 180kDa (dog) |
| 242625_at | RSAD2 | 1.3 | 11.9 | 0.000 | 1.92 | **0.028** | radical S-adenosyl methionine domain containing 2 |
| 204198_s_at | RUNX3 | 1.2 | 2.1 | 0.014 | 1.47 | 0.062 | runt-related transcription factor 3 |
| 204351_at | S100P | 1.7 | 13.3 | 0.002 | 2.50 | 0.117 | S100 calcium binding protein P |
| 205942_s_at | SAH | 1.3 | 1.0 | 0.006 | 1.36 | 0.576 | SA hypertension-associated homolog (rat) |
| 236782_at | SAMD3 | 1.6 | 4.3 | 0.019 | 1.67 | 0.995 | sterile alpha motif domain containing 3 |
| 215495_s_at | SAMD4 | -1.2 | 1.9 | 0.019 | 2.22 | **0.002** | sterile alpha motif domain containing 4 |
| 219691_at | SAMD9 | 1.4 | 2.8 | 0.001 | 1.57 | 0.447 | sterile alpha motif domain containing 9 |
| 204502_at | SAMHD1 | -1.3 | 2.2 | 0.013 | -1.09 | 0.191 | SAM domain and HD domain 1 |
| 213988_s_at | SAT | 1.3 | 2.5 | 0.024 | 1.21 | 0.128 | spermidine/spermine N1-acetyltransferase |
| 205241_at | SCO2 | 1.9 | 3.0 | 0.001 | 2.10 | 0.250 | SCO cytochrome oxidase deficient homolog 2 (yeast) |
| 205695_at | SDS | -1.0 | 1.9 | 0.006 | 1.12 | **0.042** | serine dehydratase |
| 204563_at | SELL | 1.4 | 14.7 | 0.001 | 2.41 | 0.461 | selectin L (lymphocyte adhesion molecule 1) |
| 209879_at | SELPLG | 1.0 | 3.2 | 0.009 | 1.77 | **0.015** | selectin P ligand |
| 202833_s_at | SERPINA1 | 2.0 | 5.1 | 0.003 | 2.00 | 0.287 | serine (or cysteine) proteinase inhibitor, clade A (alpha-1 antiproteinase, antitrypsin), member 1 |
| 213572_s_at | SERPINB1 | 1.5 | 3.0 | 0.005 | 1.33 | 0.303 | serine (or cysteine) proteinase inhibitor, clade B (ovalbumin), member 1 |
| 209723_at | SERPINB9 | 1.7 | 4.3 | 0.001 | 1.65 | 0.394 | serine (or cysteine) proteinase inhibitor, clade B (ovalbumin), member 9 |
| 200986_at | SERPING1 | -1.9 | 1.4 | 0.000 | -1.40 | **0.028** | serine (or cysteine) proteinase inhibitor, clade G (C1 inhibitor), member 1, (angioedema, hereditary) |
| 204019_s_at | SH3YL1 | -1.4 | -2.3 | 0.006 | -1.57 | 0.453 | SH3 domain containing, Ysc84-like 1 (S. cerevisiae) |
| 219734_at | SIDT1 | 1.1 | 1.7 | 0.006 | 1.18 | 0.507 | SID1 transmembrane family, member 1 |
| 203760_s_at | SLA | 1.2 | 4.2 | 0.000 | 1.67 | 0.079 | Src-like-adaptor /// Src-like-adaptor |
| 214734_at | SLAC2-B | -1.8 | -2.4 | 0.028 | -2.07 | 0.157 | SLAC2-B |
| 206181_at | SLAMF1 | 1.3 | 2.1 | 0.013 | 1.29 | 0.327 | signaling lymphocytic activation molecule family member 1 |
| 219159_s_at | SLAMF7 | 1.3 | 6.6 | 0.024 | 1.49 | 0.706 | SLAM family member 7 |
| 219386_s_at | SLAMF8 | 1.5 | 4.2 | 0.012 | 1.77 | 0.073 | SLAM family member 8 |
| 219593_at | SLC15A3 | 1.0 | 5.2 | 0.024 | 2.93 | **0.029** | solute carrier family 15, member 3 |
| 230748_at | SLC16A6 /// LOC440459 | 1.7 | 2.4 | 0.008 | 1.64 | 0.345 | solute carrier family 16 (monocarboxylic acid transporters), member 6 /// similar to solute carrier family 16, member 6; monocarboxylate transporter 6 |
| 201920_at | SLC20A1 | 1.9 | 4.5 | 0.016 | 1.68 | 0.823 | solute carrier family 20 (phosphate transporter), member 1 |
| 205896_at | SLC22A4 | -1.1 | 1.4 | 0.023 | 1.25 | **0.046** | solute carrier family 22 (organic cation transporter), member 4 |
| 212085_at | SLC25A6 | -1.3 | -1.6 | 0.010 | -1.12 | 0.609 | solute carrier family 25 (mitochondrial carrier; adenine nucleotide translocator), member 6 |
| 202499_s_at | SLC2A3 | 1.4 | 2.8 | 0.022 | 1.49 | 0.811 | solute carrier family 2 (facilitated glucose transporter), member 3 |
| 204588_s_at | SLC7A7 | -1.0 | 2.8 | 0.000 | 1.33 | **0.010** | solute carrier family 7 (cationic amino acid transporter, y+ system), member 7 |
| 219229_at | SLCO3A1 | -1.6 | -2.1 | 0.041 | -1.73 | 0.323 | solute carrier organic anion transporter family, member 3A1 |
| 214536_at | SLURP1 | -1.2 | -2.7 | 0.040 | -1.41 | **0.033** | secreted LY6/PLAUR domain containing 1 |
| 212569_at | SMCHD1 | 1.1 | 2.3 | 0.011 | -1.01 | 0.774 | structural maintenance of chromosomes flexible hinge domain containing 1 |
| 219519_s_at | SN | -1.0 | 8.1 | 0.002 | 1.21 | 0.087 | sialoadhesin /// sialoadhesin |
| 204466_s_at | SNCA | -1.7 | -2.5 | 0.002 | -1.82 | 0.416 | synuclein, alpha (non A4 component of amyloid precursor) /// synuclein, alpha (non A4 component of amyloid precursor) |
| 210001_s_at | SOCS1 | 1.9 | 4.1 | 0.000 | 1.40 | 0.708 | suppressor of cytokine signaling 1 |
| 216841_s_at | SOD2 | 1.9 | 4.3 | 0.005 | 1.71 | 0.906 | superoxide dismutase 2, mitochondrial |
| 228038_at | SOX2 | 1.4 | -1.0 | 0.047 | 1.46 | 0.166 | SRY (sex determining region Y)-box 2 |
| 223980_s_at | SP110 | 1.1 | 2.6 | 0.003 | 1.47 | **0.011** | SP110 nuclear body protein |
| 225564_at | SPATA13 | -1.1 | 2.4 | 0.022 | 1.02 | 0.903 | spermatogenesis associated 13 |
| 230261_at | ST8SIA4 | 1.5 | 4.1 | 0.007 | 1.47 | 0.974 | ST8 alpha-N-acetyl-neuraminide alpha-2,8-sialyltransferase 4 |
| AFFX-HUMISGF3A/M97935_5_at | STAT1 | 1.5 | 4.3 | 0.002 | 1.827663 | 0.157 | signal transducer and activator of transcription 1, 91kDa |
| 206118_at | STAT4 | 1.1 | 2.5 | 0.005 | 1.33 | 0.507 | signal transducer and activator of transcription 4 |
| 208079_s_at | STK6 | 1.7 | 2.5 | 0.004 | 1.51 | 0.771 | serine/threonine kinase 6 |
| 224724_at | SULF2 | 1.2 | 1.8 | 0.046 | 1.47 | 0.620 | sulfatase 2 |
| 213888_s_at | T3JAM | 1.5 | 3.3 | 0.014 | 1.64 | 0.164 | TRAF3-interacting Jun N-terminal kinase (JNK)-activating modulator |
| 242388_x_at | TAGAP | 1.1 | 5.4 | 0.013 | 1.33 | 0.909 | T-cell activation GTPase activating protein |
| 202307_s_at | TAP1 | -1.1 | 1.9 | 0.001 | 1.12 | 0.233 | transporter 1, ATP-binding cassette, sub-family B (MDR/TAP) |
| 225973_at | TAP2 | 1.3 | 2.1 | 0.029 | 1.47 | 0.411 | transporter 2, ATP-binding cassette, sub-family B (MDR/TAP) |
| 204158_s_at | TCIRG1 | 1.9 | 8.9 | 0.003 | 4.35 | **0.021** | T-cell, immune regulator 1, ATPase, H+ transporting, lysosomal V0 protein a isoform 3 |
| 204043_at | TCN2 | -1.0 | 2.0 | 0.001 | 1.57 | **0.034** | transcobalamin II; macrocytic anemia |
| 205943_at | TDO2 | 2.0 | 3.5 | 0.014 | 1.64 | 0.757 | tryptophan 2,3-dioxygenase |
| 206715_at | TFEC | 1.2 | 4.1 | 0.000 | 1.59 | 0.404 | transcription factor EC |
| 209277_at | TFPI2 | -1.1 | 1.7 | 0.037 | 1.25 | 0.220 | Tissue factor pathway inhibitor 2 |
| 228186_s_at | THSD2 | 1.2 | 1.5 | 0.019 | 1.67 | **0.015** | thrombospondin, type I, domain containing 2 |
| 201666_at | TIMP1 | 1.0 | 2.8 | 0.007 | 1.52 | **0.032** | tissue inhibitor of metalloproteinase 1 (erythroid potentiating activity, collagenase inhibitor) |
| 204924_at | TLR2 | 1.4 | 4.6 | 0.004 | 2.31 | 0.587 | toll-like receptor 2 |
| 206271_at | TLR3 | -1.1 | 1.5 | 0.001 | -1.03 | **0.022** | toll-like receptor 3 |
| 224341_x_at | TLR4 | -1.2 | 1.9 | 0.028 | 1.19 | 0.178 | toll-like receptor 4 /// toll-like receptor 4 |
| 229560_at | TLR8 | 1.1 | 4.2 | 0.012 | 1.68 | 0.099 | toll-like receptor 8 |
| 222735_at | TMEM38B | 1.2 | 1.5 | 0.045 | 1.09 | 0.586 | transmembrane protein 38B |
| 202643_s_at | TNFAIP3 | 1.3 | 2.4 | 0.036 | 1.17 | 0.131 | tumor necrosis factor, alpha-induced protein 3 |
| 209295_at | TNFRSF10B | 1.9 | 2.6 | 0.003 | 1.95 | 0.084 | tumor necrosis factor receptor superfamily, member 10b |
| 203508_at | TNFRSF1B | 1.2 | 4.0 | 0.013 | 1.39 | 0.121 | tumor necrosis factor receptor superfamily, member 1B |
| 202687_s_at | TNFSF10 | 1.2 | 3.1 | 0.005 | 1.67 | 0.986 | tumor necrosis factor (ligand) superfamily, member 10 /// tumor necrosis factor (ligand) superfamily, member 10 |
| 223502_s_at | TNFSF13B | 1.5 | 4.3 | 0.000 | 1.80 | **0.019** | tumor necrosis factor (ligand) superfamily, member 13b |
| 207196_s_at | TNIP1 | 1.2 | 1.5 | 0.002 | 1.37 | 0.273 | TNFAIP3 interacting protein 1 |
| 201291_s_at | TOP2A | 1.6 | 2.1 | 0.026 | 1.48 | 0.256 | topoisomerase (DNA) II alpha 170kDa |
| 224836_at | TP53INP2 | 1.8 | 2.6 | 0.042 | 2.17 | **0.047** | tumor protein p53 inducible nuclear protein 2 |
| 217143_s_at | TRA@ /// TRD@ | 1.6 | 6.9 | 0.000 | 1.96 | **0.043** | T cell receptor alpha locus /// T cell receptor delta locus |
| 215806_x_at | TRGC2 /// TRGV9 /// LOC442532 /// TARP | -1.1 | 3.1 | 0.000 | 1.33 | **0.017** | T cell receptor gamma constant 2 /// T cell receptor gamma variable 9 /// similar to T-cell receptor gamma chain C region PT-gamma-1/2 /// TCR gamma alternate reading frame protein |
| 203148_s_at | TRIM14 | 1.4 | 2.1 | 0.003 | 1.69 | 0.132 | tripartite motif-containing 14 |
| 204804_at | TRIM21 | -1.0 | 1.9 | 0.000 | 1.19 | 0.100 | tripartite motif-containing 21 |
| 213293_s_at | TRIM22 | 1.5 | 4.7 | 0.001 | 2.48 | 0.134 | tripartite motif-containing 22 |
| 221044_s_at | TRIM34 /// TRIM6-TRIM34 | -1.3 | 1.3 | 0.025 | 1.07 | 0.309 | tripartite motif-containing 34 /// tripartite motif-containing 6 and tripartite motif-containing 34 |
| 203610_s_at | TRIM38 | 1.1 | 2.6 | 0.012 | 1.30 | **0.030** | tripartite motif-containing 38 |
| 210705_s_at | TRIM5 | 1.0 | 2.1 | 0.021 | 1.36 | 0.098 | tripartite motif-containing 5 |
| 219474_at | TTMP | -1.9 | -3.1 | 0.049 | -2.57 | **0.012** | TPA-induced transmembrane protein |
| 209372_x_at | TUBB2 /// RP11-506K6.1 | 1.6 | -1.1 | 0.028 | 1.19 | 0.121 | tubulin, beta 2 /// tubulin, beta polypeptide paralog |
| 203008_x_at | TXNDC9 | 1.6 | 1.4 | 0.048 | 1.21 | **0.004** | thioredoxin domain containing 9 |
| 224511_s_at | TXNL5 | 1.9 | 2.4 | 0.002 | 1.80 | 0.399 | thioredoxin-like 5 /// thioredoxin-like 5 |
| 204122_at | TYROBP | 1.1 | 4.2 | 0.000 | 1.74 | **0.011** | TYRO protein tyrosine kinase binding protein |
| 211764_s_at | UBE2D1 | 1.8 | 2.2 | 0.010 | 1.60 | 0.642 | ubiquitin-conjugating enzyme E2D 1 (UBC4/5 homolog, yeast) /// ubiquitin-conjugating enzyme E2D 1 (UBC4/5 homolog, yeast) |
| 201649_at | UBE2L6 | 1.0 | 2.5 | 0.000 | 1.56 | **0.014** | ubiquitin-conjugating enzyme E2L 6 |
| 201535_at | UBL3 | -1.2 | -1.7 | 0.004 | -1.33 | 0.062 | ubiquitin-like 3 |
| 208998_at | UCP2 | 1.5 | 4.5 | 0.009 | 2.23 | **0.033** | uncoupling protein 2 (mitochondrial, proton carrier) |
| 219211_at | USP18 | 1.3 | 3.8 | 0.001 | 1.36 | 0.698 | ubiquitin specific protease 18 |
| 215729_s_at | VGLL1 | -1.1 | 1.5 | 0.032 | 1.21 | 0.544 | vestigial like 1 (Drosophila) |
| 200629_at | WARS | 1.3 | 3.1 | 0.002 | 1.23 | 0.335 | tryptophanyl-tRNA synthetase |
| 202664_at | WASPIP | 1.1 | 3.1 | 0.000 | 1.74 | 0.064 | Wiskott-Aldrich syndrome protein interacting protein |
| 201296_s_at | WSB1 | -1.1 | 1.4 | 0.006 | 1.16 | **0.032** | WD repeat and SOCS box-containing 1 |
| 208087_s_at | ZBP1 | 1.1 | 6.8 | 0.004 | 1.21 | 0.417 | Z-DNA binding protein 1 /// Z-DNA binding protein 1 |
| 220104_at | ZC3HAV1 | -1.0 | 2.5 | 0.002 | 1.34 | **0.032** | zinc finger CCCH type, antiviral 1 |
| 218543_s_at | ZC3HDC1 | 1.1 | 3.2 | 0.000 | 1.54 | **0.026** | zinc finger CCCH type domain containing 1 |
| 239296_at | ZFHX1B | 1.1 | 1.9 | 0.035 | 1.58 | **0.042** | Zinc finger homeobox 1b |
| 219540_at | ZNF267 | 1.7 | 2.9 | 0.003 | 1.48 | 0.682 | zinc finger protein 267 |

Abbreviations: AK — actinic keratosis, IMIQ —imiquimod, FC — fold change, Post

^1^FC AK, FC IMIQ and FC Post = median fold change for 13 subjects determined with respect to sun unexposed non lesional skin samples for pretreatment AK, imiquimod treatment and 4 weeks post imiquimod treatment respectively. The FC value for imiquimod treatment is the maximum fold change due to imiquimod treatment selected from week 1, week 2 and week 4 treatments.

^2^P-values for 2-way subject-controlled ANOVA analysis for subjects treated with imiquimod (n = 13) for comparison of imiquimod treatment (IMIQ) with pretreatment AK (AK) (see Materials and Methods section).

^3^P-values for 2-way subject-controlled ANOVA analysis for subjects treated with imiquimod (n = 13) for comparison of pretreatment AK (AK) with 4 weeks post treatment with imiquimod (Post) .

P values for 4 WK post Vs AK that are <0.05 are in bold.
