# Supplementary material for: Immune-mediated changes in actinic keratosis following topical treatment with imiquimod 5% cream
Source: J Transl Med. 2007 Jan 26;5:7. doi: 10.1186/1479-5876-5-7 (PMC1796543; doi:10.1186/1479-5876-5-7)
Supplement: Additional file 2 — Comparison of differential expression as measured by Affymetrix gene array and real time RT-PCR. This file summarizes a comparison of differential gene expression as measured by Affymetrix gene chip analysis and real time RT-PCR using low density TaqMan arrays. Expression changes for several toll-like receptors and other selected genes before and after treatment with imiquimod are reported. [file 1479-5876-5-7-S2.doc]

| **Additional file 2. Comparison of differential expression as measured by affymetrix gene array and real time RT-PCR** | | | | | | | | | | |
| --- | --- | --- | --- | --- | --- | --- | --- | --- | --- | --- |
| **Affymetrix Gene Chip** | | | | | | **Real Time RT-PCR** | | | | |
| **Gene Symbol** | **FC AK^1^** | **FC IMIQ^1^** | **P-value^2,3^** | **FC Post^1^** | **P-value^2,4^** | **FC AK^1^** | **FC IMIQ^1^** | **P-value^2,3^** | **FC Post^1^** | **P-value^2,4^** |
| *TLR1* | 1.3 | 1.8 | 0.054 | 1.2 | 0.6 | 1.1 | 2.1 | 0.010 | 1.6 | 0.552 |
| *TLR10* | NS | NS | NS | NS | NS | NS | NS | NS | NS | NS |
| *TLR2* | 1.4 | 4.6 | 0.004 | 2.3 | 0.587 | NM | NM | NM | NM | NM |
| *TLR3* | -1.1 | 1.5 | 0.001 | -1.0 | 0.022 | -2.1 | 1.5 | 0.002 | -1.3 | 0.018 |
| *TLR4* | -1.2 | 1.9 | 0.028 | 1.2 | 0.178 | -2.3 | 1.6 | 0.066 | -1.6 | 0.078 |
| *TLR5* | NS | NS | NS | NS | NS | NS | NS | NS | NS | NS |
| *TLR6* | NS | NS | NS | NS | NS | -1.1 | 1.9 | 0.037 | 1.4 | 0.210 |
| *TLR7* | 1.4 | 4.9 | 0.000 | 1.5 | 0.14 | -1.0 | 4.9 | 0.005 | 1.6 | 0.125 |
| *TLR8* | 1.1 | 4.2 | 0.012 | 1.7 | 0.099 | -1.8 | 2.4 | 0.006 | -1.5 | 0.206 |
| *TLR9* | NS | NS | NS | NS | NS | 2.9 | 15.2 | 0.041 | 4.0 | 0.287 |
| *IRF7* | 1.8 | 7.6 | 0.000 | 2.2 | 0.027 | 1.5 | 6.4 | 0.000 | 2.4 | 0.015 |
| *MyD88* | NS^5^ | NS^5^ | NS^5^ | NS^5^ | NS^5^ | 1.28 | 2.30 | 0.026 | 1.34 | 0.843 |
| *ISG15* | 1.4 | 28.3 | 0.000 | 2.1 | 0.032 | -2.15 | 2.20 | 0.006 | -1.67 | 0.341 |
| *CCL5* | 1.0 | 4.7 | 0.002 | 1.4 | 0.026 | 1.25 | 8.49 | 0.001 | 2.61 | 0.117 |
| *CD80* | NS | NS | NS | NS | NS | 1.79 | 10.53 | 0.015 | 1.54 | 0.895 |
| *CD86* | 1.1 | 2.9 | 0.000 | 1.5 | 0.020 | -1.37 | 2.76 | 0.000 | -1.02 | 0.138 |
| *CXCL10* | 1.5 | 12.6 | 0.002 | 2.6 | 0.253 | 3.93 | 72.12 | 0.025 | 4.15 | 0.337 |
| *GBP2* | 1.0 | 1.7 | 0.012 | 1.2 | 0.294 | -1.95 | 1.22 | 0.009 | -1.02 | 0.046 |

Abbreviations: NS— statistically not significant, NM — not measured, FC — fold change, AK — actinic keratosis, Post — post treatment, IMIQ — imiquimod, RT‑PCR — reverse transcriptase polymerase chain reaction.

^1^FC = median fold change for 13 subjects.

^2^P-values for 2-way subject-controlled ANOVA analysis for subjects treated with imiquimod (n = 13).

^3^P values for comparison of imiquimod treatment (maximum fold change due to imiquimod treatment selected from week 1, week 2 and week 4 treatments) with pretreatment AK (see Materials and Methods section).

^4^P values for comparison of 4-week post treatment samples with pretreatment AK samples.

^5^MyD88 was found to be differentially expressed in the Affymetrix analysis but was not included in the ANOVA analysis because it did not meet the criteria of at least 1 sample showing a fold change value of >4.
