# Supplementary material for: Immune-mediated changes in actinic keratosis following topical treatment with imiquimod 5% cream
Source: J Transl Med. 2007 Jan 26;5:7. doi: 10.1186/1479-5876-5-7 (PMC1796543; doi:10.1186/1479-5876-5-7)
Supplement: Additional file 3 — Imiquimod-induced genes with immune response gene ontology category and/or known to be IFNα/β-inducible. This file reports on expression changes before and after treatment with imiquimod, for selected genes with gene ontology category of immune response and (or) are know to be inducible by type 1 interferons. [file 1479-5876-5-7-S3.doc]

| **Additional file 3. Imiquimod-induced genes with immune response gene ontology category and/or known to be IFNα/β-inducible** | | | | | | | | |
| --- | --- | --- | --- | --- | --- | --- | --- | --- |
| **Gene Symbol** | **FC AK^1^** | **FC IMIQ^1^** | **P-value^2^** | **Gene Title** | **GO Cellular Process^3^** | **Immune Response^4^** | **IFN-inducible^5^** | **Reference^6^** |
| *AIM2* | 1.5 | 2.9 | 0.002 | absent in melanoma 2 | immune response | Yes | Yes | 2, 6 |
| *APOL3* | 1.0 | 2.1 | 0.011 | apolipoprotein L, 3 | lipid transport | Yes | Yes | 2 |
| *BST2* | -1.1 | 11.2 | 0.000 | bone marrow stromal cell antigen 2 | humoral immune response | Yes | Yes | 2, 4, 5, 6 |
| *CCL5* | 1.1 | 19.4 | 0.001 | chemokine (C-C motif) ligand 5, *RANTES* | chemotaxis, cell adhesion | Yes | Yes | 6 |
| *CCL8* | 1.5 | 10.7 | 0.000 | chemokine (C-C motif) ligand 8 | inflammatory response | Yes | Yes | 2 |
| *CCR1* | 1.2 | 9.3 | 0.000 | chemokine (C-C motif) receptor 1 | chemotaxis | Yes | Yes | 2, 4, 6 |
| *CD164* | 1.4 | 2.2 | 0.025 | CD164 antigen, sialomucin | negative regulation of cell adhesion and cell proliferation | Yes | Yes | 2, 4 |
| *CD74* | 1.1 | 1.6 | 0.048 | CD74 antigen | antigen presentation | Yes | Yes | 6 |
| *CD8B1* | 1.0 | 1.4 | 0.018 | CD8 antigen, beta polypeptide 1 | T-cell activation | Yes | Yes | 3 |
| *CEACAM1* | -1.2 | 1.5 | 0.001 | carcinoembryonic antigen-related cell adhesion molecule 1 | immune response | Yes | Yes | 1 |
| *CTSS* | 1.2 | 3.2 | 0.001 | cathepsin S | proteolysis and peptidolysis | Yes | Yes | 6 |
| *CXCL10* | 1.5 | 12.6 | 0.002 | chemokine (C-X-C motif) ligand 10 | chemotaxis | Yes | Yes | 1, 2, 3, 4, 6 |
| *CXCL11* | 1.8 | 40.8 | 0.001 | chemokine (C-X-C motif) ligand 11, *ITAC* | chemotaxis | Yes | Yes | 2, 6 |
| *EIF2AK2* | 1.1 | 3.8 | 0.000 | eukaryotic translation initiation factor 2-alpha kinase 2 | regulation of transcription | Yes | Yes | 1, 2, 4, 5, 6 |
| *FCER1A* | -1.1 | -2.2 | 0.017 | receptor for Fc fragment of IgE, high affinity I | immune response | Yes | Yes | 3 |
| *G1P2* | 1.4 | 28.3 | 0.000 | interferon, alpha-inducible protein (clone IFI-15K) | immune response | Yes | Yes | 2, 4, 5, 6 |
| *G1P3* | 1.4 | 7.1 | 0.001 | interferon, alpha-inducible protein (clone IFI-6-16) | immune response | Yes | Yes | 1, 4, 5, 6 |
| *GBP1* | 1.5 | 8.2 | 0.000 | guanylate binding protein 1 | immune response | Yes | Yes | 1, 2, 3, 6 |
| *GBP2* | 1.0 | 1.7 | 0.012 | guanylate binding protein 2 | immune response | Yes | Yes | 2, 3, 5 |
| *HLA-DPA1* | 1.5 | 2.1 | 0.041 | major histocompatibility complex, class II, DP alpha 1 | immune response | Yes | Yes | 2 |
| *IFI16* | 1.7 | 2.6 | 0.002 | interferon, gamma-inducible protein 16 | regulation of transcription | Yes | Yes | 1, 2, 5 |
| *IFI30* | 1.3 | 3.0 | 0.000 | interferon, gamma-inducible protein 30 | --- | Yes | Yes | 6 |
| *IFI35* | -1.0 | 3.8 | 0.000 | interferon-induced protein 35 | response to virus | Yes | Yes | 2, 5, 6 |
| *IFIT1* | 1.4 | 19.8 | 0.014 | interferon-induced protein with tetratricopeptide repeats | immune response | Yes | Yes | 1, 2, 3, 4, 5, 6 |
| *IFIT3* | 1.0 | 4.9 | 0.001 | interferon-induced protein with tetratricopeptide repeats 3, *RIG-G* | immune response | Yes | Yes | 2, 3, 5, 6 |
| *IFIT5* | 1.1 | 2.4 | 0.002 | interferon-induced protein with tetratricopeptide repeats 5 | immune response | Yes | Yes | 6 |
| *IFITM1* | 1.2 | 3.8 | 0.000 | interferon induced transmembrane protein 1 (9-27) | regulation of cell cycle | Yes | Yes | 3 |
| *IL21R* | 1.2 | 8.6 | 0.000 | interleukin 21 receptor | natural killer cell activation | Yes | Yes | 3 |
| *INDO* | 1.4 | 4.1 | 0.001 | indoleamine-pyrrole 2,3 dioxygenase | tryptophan catabolism | Yes | Yes | 2 |
| *IRF1* | 1.2 | 2.1 | 0.001 | interferon regulatory factor 1 | regulation of transcription | Yes | Yes | 3, 5, 6 |
| *IRF7* | 1.8 | 7.6 | 0.000 | interferon regulatory factor 7 | regulation of transcription | Yes | Yes | 2, 4, 6 |
| *ITK* | 1.4 | 1.9 | 0.022 | IL2-inducible T-cell kinase | cellular defense response | Yes | Yes | 3 |
| *KLRC1 /// KLRC2* | 1.1 | 31.6 | 0.000 | killer cell lectin-like receptor subfamily C, member 1 ///member 2 | cellular defense response | Yes | Yes | 3 |
| *LY96* | 1.2 | 4.9 | 0.000 | lymphocyte antigen 96 | antibacterial humoral response | Yes | Yes | 4 |
| *MICB* | 1.3 | 4.2 | 0.000 | MHC class I polypeptide-related sequence B | antigen presentation | Yes | Yes | 2 |
| *MX1* | 1.2 | 11.5 | 0.000 | myxovirus resistance 1, | apoptosis | Yes | Yes | 1, 2, 4, 5, 6 |
| *MX2* | 1.0 | 4.8 | 0.000 | myxovirus resistance 2 (mouse) | immune response | Yes | Yes | 2, 3, 4, 5, 6 |
| *NMI* | 1.9 | 3.0 | 0.001 | N-myc (and STAT) interactor | JAK-STAT cascade | Yes | Yes | 1, 2, 6 |
| *OAS1* | 1.9 | 6.9 | 0.000 | 2',5'-oligoadenylate synthetase 1, 40/46kDa | nucleotide and nucleic acid metabolism | Yes | Yes | 2, 4, 5 |
| *OAS2* | 1.2 | 3.7 | 0.000 | 2'-5'-oligoadenylate synthetase 2, 69/71kDa | nucleotide and nucleic acid metabolism | Yes | Yes | 2, 3, 4, 5, 6 |
| *OAS3* | 1.4 | 3.9 | 0.000 | 2'-5'-oligoadenylate synthetase 3, 100kDa | nucleotide and nucleic acid metabolism | Yes | Yes | 2, 4, 6 |
| *OASL* | 1.6 | 5.1 | 0.000 | 2'-5'-oligoadenylate synthetase-like | immune response | Yes | Yes | 2 |
| *PRF1* | 1.0 | 2.3 | 0.002 | perforin 1 (pore forming protein) | programmed cell death, cytolysis | Yes | Yes | 2 |
| *PSMB10* | 1.3 | 2.5 | 0.015 | proteasome subunit, beta type, 10 | proteolysis and peptidolysis | Yes | Yes | 2, 6 |
| *PSMB9* | 1.1 | 2.5 | 0.003 | proteasome subunit, beta type, 9 | proteolysis and peptidolysis | Yes | Yes | 2, 4, 6 |
| *SAMHD1* | -1.3 | 2.2 | 0.013 | SAM domain and HD domain 1, *DCIP* | immune response | Yes | Yes | 2, 6 |
| *SERPING1* | -1.9 | 1.4 | 0.000 | serine (or cysteine) proteinase inhibitor, clade G member 1 | complement activation | Yes | Yes | 2, 6 |
| *SN* | -1.0 | 8.1 | 0.002 | sialoadhesin | cell-cell adhesion | Yes | Yes | 2, 3 |
| *TAP1* | -1.1 | 1.9 | 0.001 | transporter 1, ATP-binding cassette, sub-family B (*MDR/TAP*) | oligopeptide transport | Yes | Yes | 2, 5, 6 |
| *TLR3* | -1.1 | 1.5 | 0.001 | Toll-like receptor 3 | interferon-beta biosynthesis | Yes | Yes | 1, 6 |
| *TLR7* | 1.4 | 4.9 | 0.000 | Toll-like receptor 7 | immune response | Yes | Yes | 7 |
| *TNFSF10* | 1.2 | 3.1 | 0.005 | tumor necrosis factor (ligand) superfamily, member 10 | apoptosis | Yes | Yes | 1, 2, 6 |
| *AKAP2* | -1.3 | 1.7 | 0.018 | A kinase (PRKA) anchor protein 2 | --- | --- | Yes | 2 |
| *ATF5* | 1.1 | 1.7 | 0.001 | activating transcription factor 5 | regulation of cell cycle | --- | Yes | 4 |
| *BIRC3* | 1.9 | 3.8 | 0.012 | baculoviral IAP repeat-containing 3 | anti-apoptosis | --- | Yes | 1 |
| *BTN3A3* | -1.1 | 2.5 | 0.016 | butyrophilin, subfamily 3, member A3 | --- | --- | Yes | 1, 2 |
| *C1orf38* | 1.2 | 3.1 | 0.009 | chromosome 1 open reading frame 38 | cell adhesion | --- | Yes | 1 |
| *C20orf18* | -1.4 | 1.4 | 0.027 | Chromosome 20 open reading frame 18 | protein ubiquitination | --- | Yes | 2, 6 |
| *CASP1* | 1.4 | 2.1 | 0.009 | caspase 1, apoptosis-related cysteine protease | regulation of apoptosis | --- | Yes | 1, 2 |
| *CCR5* | 2.0 | 5.0 | 0.005 | chemokine (C-C motif) receptor 5 | G-protein coupled receptor | --- | Yes | 2 |
| *CD38* | 1.3 | 7.5 | 0.000 | CD38 antigen (p45) | apoptosis | --- | Yes | 2 |
| *CD47* | 1.8 | 2.6 | 0.041 | CD47 antigen (integrin-associated signal transducer) | cell-matrix adhesion | --- | Yes | 2 |
| *CD69* | 1.8 | 6.3 | 0.046 | CD69 antigen (p60, early T-cell activation antigen) | defense response | --- | Yes | 2 |
| *CECR1* | 1.5 | 3.8 | 0.012 | cat eye syndrome chromosome region, candidate 1 | development | --- | Yes | 2 |
| *DDX58* | 1.4 | 4.1 | 0.004 | DEAD (Asp-Glu-Ala-Asp) box polypeptide 58, *RIG-I* | --- | --- | Yes | 1, 2, 4, 6 |
| *ECGF1* | 1.6 | 2.9 | 0.033 | endothelial cell growth factor 1 | cell differentiation | --- | Yes | 2 |
| *ENPP2* | -1.1 | 2.1 | 0.027 | Ectonucleotide pyrophosphatase/phosphodiesterase 2 (autotaxin) | chemotaxis | --- | Yes | 2 |
| *GCH1* | 1.5 | 2.8 | 0.011 | GTP cyclohydrolase 1 (dopa-responsive dystonia) | nitric oxide biosynthesis | --- | Yes | 2 |
| *HERC5* | 1.0 | 3.4 | 0.000 | hect domain and *RLD 5* | ubiquitin cycle | --- | Yes | 3, 4 |
| *HLA-E* | -1.8 | -1.1 | 0.004 | major histocompatibility complex, class I, E | antigen presentation | --- | Yes | 2, 6 |
| *HSXIAPAF1* | 1.3 | 6.3 | 0.000 | XIAP associated factor-1 | --- | --- | Yes | 2, 6 |
| *IFI44* | 1.7 | 12.6 | 0.000 | interferon-induced protein 44 | response to virus | --- | Yes | 2, 4, 5, 6 |
| *IFI44L* | 1.0 | 16.3 | 0.096 | interferon-induced protein 44-like | --- | --- | Yes | 4 |
| *IFIH1* | 1.2 | 5.7 | 0.022 | interferon induced with helicase C domain 1, MDA5 | regulation of apoptosis | --- | Yes | 2, 6 |
| *IFIT2* | -1.2 | 7.4 | 0.000 | interferon-induced protein with tetratricopeptide repeats 2 | immune response | --- | Yes | 2, 4, 5, 6 |
| *ISG20* | 1.3 | 6.1 | 0.000 | interferon stimulated gene 20kDa | response to virus | --- | Yes | 2, 3, 4, 6 |
| *ITGA4* | 1.5 | 7.4 | 0.022 | integrin, alpha 4 (CD49D, alpha 4 subunit of VLA-4 receptor) | cell-matrix adhesion | --- | Yes | 3 |
| *LAG3* | -1.1 | 4.9 | 0.000 | lymphocyte-activation gene 3 | --- | --- | Yes | 2 |
| *LAMP3* | 1.6 | 2.5 | 0.008 | lysosomal-associated membrane protein 3 | cell proliferation | --- | Yes | 2, 6 |
| *LAP3* | 1.4 | 2.4 | 0.001 | leucine aminopeptidase 3 | proteolysis and peptidolysis | --- | --- | 2, 4 |
| *LBA1* | 1.2 | 1.7 | 0.000 | lupus brain antigen 1 | --- | --- | Yes | 1 |
| *LGALS9* | 1.6 | 5.6 | 0.011 | lectin, galactoside-binding, soluble, 9 (galectin 9) | positive regulation of I-kappaB kinase/NF-kappaB cascade | --- | Yes | 1, 2, 6 |
| *LGP2* | -1.1 | 2.2 | 0.002 | likely ortholog of mouse D11lgp2 | --- | --- | Yes | 2 |
| *LOC129607* | 1.3 | 11.8 | 0.002 | hypothetical protein LOC129607 | --- | --- | Yes | 4 |
| *LY6E* | 1.1 | 2.7 | 0.002 | lymphocyte antigen 6 complex, locus E | defense response | --- | Yes | 2, 6 |
| *NKG7* | 1.2 | 9.6 | 0.001 | natural killer cell group 7 | --- | --- | Yes | 4 |
| *NR4A3* | 1.6 | 2.3 | 0.043 | nuclear receptor subfamily 4, group A, member 3 | regulation of transcription | --- | Yes | 2 |
| *PARP14* | 1.5 | 3.7 | 0.000 | poly (ADP-ribose) polymerase family, member 14 | protein amino acid ADP-ribosylation | --- | Yes | 4 |
| *PHLDA1* | 1.6 | 2.2 | 0.032 | pleckstrin homology-like domain, family A, member 1 | --- | --- | Yes | 3 |
| *PLAC8* | 1.7 | 11.0 | 0.001 | placenta-specific 8 | --- | --- | Yes | 2 |
| *PLSCR1* | 1.5 | 5.3 | 0.001 | phospholipid scramblase 1 | phospholipid scrambling | --- | Yes | 2, 5, 6 |
| *PML* | -1.1 | 4.5 | 0.002 | promyelocytic leukemia | protein ubiquitination | --- | Yes | 2, 6 |
| *PSME2* | 1.4 | 2.0 | 0.007 | proteasome (prosome, macropain) activator subunit 2 (PA28 beta) | immune response | --- | Yes | 1, 2, 4 |
| *RARRES3* | -1.2 | 2.9 | 0.001 | retinoic acid receptor responder (tazarotene induced) 3 | negative regulation of cell proliferation | --- | Yes | 2 |
| *RSAD2* | 1.3 | 11.9 | 0.000 | radical S-adenosyl methionine | domain containing 2, Cig5 | --- | Yes | 2, 3, 4, 6 |
| *SAT* | 1.3 | 2.5 | 0.024 | spermine N1-acetyltransferase | --- | --- | Yes | 2 |
| *SCO2* | 1.9 | 3.0 | 0.001 | SCO cytochrome oxidase deficient homolog 2 (yeast) | electron transport | --- | Yes | 2 |
| *SERPINB1* | 1.5 | 3.0 | 0.005 | serine (or cysteine) proteinase inhibitor, | clade B (ovalbumin), member 1 | --- | Yes | 2 |
| *SOCS1* | 1.9 | 4.1 | 0.000 | suppressor of cytokine signaling 1 | ubiquitin cycle, regulation of cell growth | --- | Yes | 2 |
| *SP110* | 1.1 | 2.6 | 0.003 | SP110 nuclear body protein | regulation of transcription | --- | Yes | 1, 2, 4, 6 |
| *STAT1* | 1.5 | 4.3 | 0.002 | signal transducer and activator of transcription 1, 91kDa | --- | --- | Yes | 1, 2, 3, 4, 5 |
| *TDO2* | 2.0 | 3.5 | 0.014 | tryptophan 2,3-dioxygenase | tryptophan metabolism | --- | Yes | 1 |
| *TFEC* | 1.2 | 4.1 | 0.000 | transcription factor EC | --- | --- | Yes | 2 |
| *TNFAIP3* | 1.3 | 2.4 | 0.036 | tumor necrosis factor, alpha-induced protein 3 | ubiquitin cycle, anti-apoptosis | --- | Yes | 2 |
| *TNFSF13B* | 1.5 | 4.3 | 0.000 | tumor necrosis factor (ligand) superfamily, member 13b | cell proliferation | --- | Yes | 3 |
| *TRIM14* | 1.4 | 2.1 | 0.003 | tripartite motif-containing 14 | compartment specification | --- | Yes | 2, 5 |
| *TRIM22* | 1.5 | 4.7 | 0.001 | tripartite motif-containing 22, *STAF50* | protein ubiquitination | --- | Yes | 1, 2, 3, 4, 5, 6 |
| *TRIM34* | -1.3 | 1.3 | 0.025 | tripartite motif-containing 34 | protein ubiquitination | --- | Yes | 1, 2 |
| *TRIM38* | 1.1 | 2.6 | 0.012 | tripartite motif-containing 38 | protein ubiquitination | --- | Yes | 2 |
| *TRIM5* | 1.0 | 2.1 | 0.021 | tripartite motif-containing 5 | protein ubiquitination | --- | Yes | 2, 6 |
| *UBE2L6* | 1.0 | 2.5 | 0.000 | ubiquitin-conjugating enzyme E2L 6 | ubiquitin cycle | --- | Yes | 2, 4, 6 |
| *USP18* | 1.3 | 3.8 | 0.001 | ubiquitin specific protease 18 | ubiquitin cycle | --- | Yes | 2, 4, 6 |
| *WARS* | 1.3 | 3.1 | 0.002 | tryptophanyl-tRNA synthetase | protein biosynthesis | --- | Yes | 2 |
| *WASPIP* | 1.1 | 3.1 | 0.000 | Wiskott-Aldrich syndrome protein interacting protein | protein complex assembly | --- | Yes | 2 |
| *ZBP1* | 1.1 | 6.8 | 0.004 | Z-DNA binding protein 1 | --- | --- | Yes | 2 |
| *ZNF267* | 1.7 | 2.9 | 0.003 | zinc finger protein 267 | regulation of transcription, DNA-dependent | --- | Yes | 2 |
| *AIF1* | 1.2 | 4.9 | 0.022 | allograft inflammatory factor 1 | cell cycle arrest | Yes | --- | --- |
| *APOL2* | 1.8 | 4.7 | 0.004 | apolipoprotein L, 2 | acute-phase response | Yes | --- | --- |
| *C1QA* | 1.0 | 2.8 | 0.000 | complement component 1, q subcomponent, alpha polypeptide | complement activation | Yes | --- | --- |
| *C1QB* | 1.1 | 4.1 | 0.001 | complement component 1, q subcomponent, beta polypeptide | complement activation | Yes | --- | --- |
| *C3AR1* | 1.0 | 2.6 | 0.024 | complement component 3a receptor 1 | inflammatory response | Yes | --- | --- |
| *C5R1* | 1.1 | 5.1 | 0.009 | complement component 5 receptor 1 (C5a ligand) | chemotaxis, cellular defense response | Yes | --- | --- |
| *CCL3* | 1.8 | 6.1 | 0.006 | chemokine (C-C motif) ligand 3, MIP1a | chemotaxis | Yes | --- | --- |
| *CD14* | 1.1 | 4.0 | 0.013 | CD14 antigen | phagocytosis, apoptosis | Yes | --- | --- |
| *CD163* | -1.0 | 5.0 | 0.000 | CD163 antigen | antimicrobial humoral response | Yes | --- | --- |
| *CD1C* | 1.1 | -6.1 | 0.005 | CD1C antigen, c polypeptide | antimicrobial humoral response | Yes | --- | --- |
| *CD2* | 1.6 | 5.0 | 0.019 | CD2 antigen (p50), sheep red blood cell receptor | apoptosis, cell-cell adhesion | Yes | --- | --- |
| *CD53* | 1.6 | 5.6 | 0.001 | CD53 antigen | antimicrobial humoral response | Yes | --- | --- |
| *CD86* | 1.1 | 2.9 | 0.000 | CD86 antigen, B7-2 antigen) | T-cell activation, costimulation | Yes | --- | --- |
| *CD97* | -1.3 | 1.3 | 0.005 | CD97 antigen | cell motility | Yes | --- | --- |
| *CLEC4A* | 1.5 | 2.3 | 0.008 | C-type (calcium dependent, carbohydrate-recognition domain) lectin, superfamily member 6 | cell adhesion | Yes | --- | --- |
| *CSF2RB* | 1.3 | 4.1 | 0.007 | colony stimulating factor 2 receptor, beta, low-affinity | antimicrobial humoral response | Yes | --- | --- |
| *CST7* | 1.2 | 2.5 | 0.029 | cystatin F (leukocystatin) | immune response | Yes | --- | --- |
| *CTSC* | 1.5 | 2.6 | 0.039 | cathepsin C | proteolysis and peptidolysis | Yes | --- | --- |
| *CXCL12* | 1.4 | 1.8 | 0.014 | chemokine (C-X-C motif) ligand 12 | calcium ion homeostasis | Yes | --- | --- |
| *CXCL5* | 1.4 | 3.4 | 0.023 | chemokine (C-X-C motif) ligand 5 | chemotaxis | Yes | --- | --- |
| *CYBB* | 1.2 | 2.6 | 0.001 | cytochrome b-245, beta polypeptide | electron transport | Yes | --- | --- |
| *EBI2* | 1.5 | 4.3 | 0.007 | Epstein-Barr virus induced gene 2 (lymphocyte-specific G protein-coupled receptor) | immune response | Yes | --- | --- |
| *FCER1G* | 1.0 | 2.9 | 0.002 | Fc fragment of IgE, high affinity I, receptor for; gamma polypeptide | immune response | Yes | --- | --- |
| *FCGR1A* | 2.0 | 12.4 | 0.007 | Fc fragment of IgG, high affinity Ia, receptor for (*CD64*) | --- | Yes | --- | --- |
| *FCGR3B* | 1.6 | 2.7 | 0.014 | Fc fragment of IgG, low affinity IIIa, receptor for (*CD16*) | immune response | Yes | --- | --- |
| *FPR1* | 1.1 | 2.6 | 0.019 | formyl peptide receptor 1 | chemotaxis | Yes | --- | --- |
| *FYB* | 1.6 | 6.5 | 0.002 | FYN binding protein (*FYB-120/130*) | protein kinase cascade | Yes | --- | --- |
| *GNLY* | 1.3 | 13.3 | 0.011 | Granulysin | cellular defense response | Yes | --- | --- |
| *GZMA* | 1.3 | 5.4 | 0.002 | granzyme A | apoptosis, cytolysis | Yes | --- | --- |
| *IGLV2-14* | 1.2 | 2.6 | 0.029 | immunoglobulin lambda joining 3 | --- | Yes | --- | --- |
| *IGSF6* | 1.6 | 6.7 | 0.001 | immunoglobulin superfamily, member 6 | immune response | Yes | --- | --- |
| *IL18RAP* | 1.1 | 3.0 | 0.001 | interleukin 18 receptor accessory protein | inflammatory response | Yes | --- | --- |
| *ILT7* | 1.2 | 5.0 | 0.001 | leukocyte immunoglobulin-like receptor, subfamily A (without TM domain), member 4, *LILRA4* | immune response | Yes | --- | --- |
| *IRF8* | 1.9 | 5.7 | 0.028 | interferon consensus sequence binding protein 1 | regulation of transcription | Yes | --- | --- |
| *ITGB2* | 1.2 | 3.1 | 0.012 | integrin, beta 2 (antigen CD18 (p95), lymphocyte function-associated antigen 1 | apoptosis, cell-matrix adhesion | Yes | --- | --- |
| *KLRF1* | 1.2 | 5.3 | 0.001 | killer cell lectin-like receptor subfamily F, member 1 | antimicrobial humoral response | Yes | --- | --- |
| *LCP2* | 1.3 | 4.9 | 0.047 | lymphocyte cytosolic protein 2 | immune response | Yes | --- | --- |
| *LGALS3BP* | 1.4 | 3.4 | 0.001 | lectin, galactoside-binding, soluble, 3 binding protein | cellular defense response, adhesion | Yes | --- | --- |
| *LILRA1* | 1.0 | 4.5 | 0.003 | leukocyte immunoglobulin-like receptor, subfamily B (with TM and ITIM domains), member 1 | immune response | Yes | --- | --- |
| *LILRB2 /// LILRB3* | -1.1 | 3.2 | 0.000 | leukocyte immunoglobulin-like receptor, subfamily B (with TM and ITIM domains), member 3 | cellular defense response | Yes | --- | --- |
| *NCF2* | -1.0 | 1.9 | 0.000 | neutrophil cytosolic factor 2 | superoxide metabolism | Yes | --- | --- |
| *SERPINA1* | 2.0 | 5.1 | 0.003 | serine (or cysteine) proteinase inhibitor, clade A (alpha-1 antiproteinase, antitrypsin), member 1 | acute-phase response | Yes | --- | --- |
| *SLAMF1* | 1.3 | 2.1 | 0.013 | signaling lymphocytic activation molecule family member - | lymphocyte activation | Yes | --- | --- |
| *TCIRG1* | 1.9 | 8.9 | 0.003 | T cell, immune regulator 1, ATPase, H+ transporting, lysosomal V0 protein a isoform 3 | cellular defense response | Yes | --- | --- |
| *TLR2* | 1.4 | 4.6 | 0.004 | Toll-like receptor 2 | apoptosis, inflammatory response | Yes | --- | --- |
| *TLR4* | -1.2 | 1.9 | 0.028 | Toll-like receptor 4 | immune response | Yes | --- | --- |
| *TLR8* | 1.1 | 4.2 | 0.012 | Toll-like receptor 8 | immune response | Yes | --- | --- |
| *TRA@ /// TRD@* | 1.6 | 6.9 | 0.000 | T cell receptor delta locus | cellular defense response | Yes | --- | --- |
| *TYROBP* | 1.1 | 4.2 | 0.000 | TYRO protein tyrosine kinase binding protein | cellular defense response | Yes | --- | --- |

Abbreviations: AK — actinic keratosis, IMIQ —imiquimod, GO — Gene Ontology, IFN — interferon, FC — fold change.

^1^FC = median fold change for 13 subjects.

^2^P-values for 2-way subject-controlled ANOVA analysis for subjects treated with imiquimod (n = 13) for comparison of imiquimod treatment with pretreatment AK (see Materials and Methods section). The value for imiquimod treatment is the maximum fold change due to imiquimod treatment selected from week 1, week 2 and week 4 treatments.

^3^GO cellular process classification from Gene Ontology.

^4^Immune response classification from GO.

^5^Interferon inducibility shown in various cellular systems in the references cited.

^6^References in table:

1. Geiss GK, Carter VS, He Y, Kwieciszewski BK, Holzman T, Korth MJ, *et al* (2003). Gene expression profiling of the cellular transcriptional network regulated by alpha/beta interferon and its partial attenuation by the hepatitis C virus nonstructural 5A protein*. J Virol* 77(11):6367-6375.

2. Taylor MW, Grosse WM, Schaley JE, Sanda C, Wu X, Chien SC, *et al* (2004). Global effect of PEG-IFN-alpha and ribavirin on gene expression in PBMC *in vitro*. *J Interferon Cytokine Res* 24(2):107-118.

3. Nagorsen D, Deola S, Smith K, Wang E, Monsurro V, Zanovello P, *et al* (2005). Polarized monocyte response to cytokine stimulation. *Genome Biol* 6(2):R15.

4. Stroncek DF, Basil C, Nagorsen D, Deola S, Arico E, Smith K, *et al* (2005). Delayed polarization of mononuclear phagocyte transcriptional program by type I interferon isoforms. *J Transl Med* 3:24.

5. Der SD, Zhou A, Williams BR, Silverman RH (1998). Identification of genes differentially regulated by interferon alpha, beta, or gamma using oligonucleotide arrays. *Proc Natl Acad Sci USA* 95(26):15623-15628.

6. Leaman DW, Chawla-Sarkar M, Jacobs B, Vyas K, Sun Y, Ozdemir A, Yi T, Williams BR, *et al* (2003). Novel growth and death related interferon-stimulated genes (ISGs) in melanoma: Greater potency of IFN-beta compared with IFN-alpha2. *J Interferon Cytokine Res* 23(12):745-756.

7 Mohty M, Vialle-Castellano A, Nunes JA, Isnardon D, Olive D, Gaugler B (2003). IFN-alpha skews monocyte differentiation into Toll-like receptor 7-expressing dendritic cells with potent functional activities. J Immunol 171(7):3385-3393.
